# SWO1 modulates cell wall integrity under salt stress by interacting with importin ɑ in Arabidopsis

**DOI:** 10.1007/s44154-021-00010-5

**Published:** 2021-09-29

**Authors:** Zhidan Wang, Mugui Wang, Changhong Yang, Lun Zhao, Guochen Qin, Li Peng, Qijie Zheng, Wenfeng Nie, Chun-Peng Song, Huazhong Shi, Jian-Kang Zhu, Chunzhao Zhao

**Affiliations:** 1 Shanghai Center for Plant Stress Biology, CAS Center for Excellence in Molecular Plant Sciences, Chinese Academy of Sciences, Shanghai, 200032 China; 2 University of Chinese Academy of Sciences, Beijing, 100049 China; 3 National Key Laboratory of Crop Genetic Improvement, Huazhong Agricultural University, Wuhan, 430070 China; 4 Department of Horticulture, College of Horticulture and Plant Protection, Yangzhou University, Yangzhou, 225009 China; 5 Key Laboratory of Plant Stress Biology, School of Life Sciences, Henan University, Kaifeng, 475001 China; 6 Department of Chemistry and Biochemistry, Texas Tech University, Lubbock, TX 79409 USA

**Keywords:** Agenet domain, Salt stress, Importins, Plant cell wall, Arabidopsis

## Abstract

Maintenance of cell wall integrity is of great importance not only for plant growth and development, but also for the adaptation of plants to adverse environments. However, how the cell wall integrity is modulated under salt stress is still poorly understood. Here, we report that a nuclear-localized Agenet domain-containing protein SWO1 (SWOLLEN 1) is required for the maintenance of cell wall integrity in Arabidopsis under salt stress. Mutation in *SWO1* gene results in swollen root tips, disordered root cell morphology, and root elongation inhibition under salt stress. The *swo1* mutant accumulates less cellulose and pectin but more lignin under high salinity. RNA-seq and ChIP-seq assays reveal that SWO1 binds to the promoter of several cell wall-related genes and regulates their expression under saline conditions. Further study indicates that SWO1 interacts with importin ɑ IMPA1 and IMPA2, which are required for the import of nuclear-localized proteins. The *impa1 impa2* double mutant also exhibits root growth inhibition under salt stress and mutations of these two genes aggravate the salt-hypersensitive phenotype of the *swo1* mutant. Taken together, our data suggest that SWO1 functions together with importin ɑ to regulate the expression of cell wall-related genes, which enables plants to maintain cell wall integrity under high salinity.

**Supplementary Information:**

The online version contains supplementary material available at 10.1007/s44154-021-00010-5.

## Introduction

Soil salinity is one of the paramount factors that limit plant distribution, growth, and yield. Currently more than one-third of irrigated lands are affected by salinization in the world (FAO, 2011), and therefore improving the salt tolerance of crops is of great importance for global food security and sustainable agriculture. Upon exposure to high salinity, plants experience dramatic morphological, physiological, biochemical, and metabolic changes in order to adapt to adverse conditions. More and more evidences have shown that maintenance of plant cell wall integrity is critical for salt tolerance, especially for root elongation under high salinity (Shi et al., 2003; Zhu et al., 2010; Zhang et al., 2016; Zhao et al., 2018a; Zhao et al., 2019b), and cell wall biosynthesis is a dynamic process, which is regulated based on broad extracellular and intracellular contexts.

The plant cell wall consists of a polysaccharide network, in which cellulose microfibrils are crosslinked by hemicelluloses in a pectin matrix. Many glycoproteins and secreted peptides are also identified in the cell wall matrix (Somerville, 2006). Maintenance of cell wall integrity is critical for cell expansion during growth and development, and also allows plants to adapt to changing environments (Anderson & Kieber, 2020). Extensive studies have shown that perturbation of cell wall integrity affects stress tolerance in plants. For example, mutation in the *SOS5* gene, which encodes an arabinogalactan protein with AGP-like and fasciclin-like domains, results in an arrested root elongation and a swollen root tip under salt stress (Shi et al., 2003). *SOS6*, encoding a cellulose synthase-like protein, is involved in the regulation of root elongation under salt stress (Zhu et al., 2010). Disruption of the key component in cellulose synthase complex, CESA6, leads to a salt-hypersensitive phenotype in Arabidopsis (Zhang et al., 2016). The companions of CESAs, including CC1 and CC2, are required for the elongation of hypocotyls under salt stress (Endler et al., 2015). A recent study showed that the receptor-like kinase FER, together with the cell wall-localized leucine-rich repeat extensins LRX3, LRX4, and LRX5, and the secretory peptides RALFs, function as a module to monitor cell wall integrity and regulate salt tolerance in Arabidopsis (Zhao et al., 2018a). *MUR4* encodes a UDP-D-xylose 4-epimerase that is required for the the biosynthesis of UDP-arabinose (UDP-Ara), and UDP-Ara participates in the decorations of several cell wall polysaccharides and glycoproteins and thereby regulates cell wall integrity. Loss of function of the *MUR4* gene leads to a reduced root elongation and abnormal cell adhesion under high salinity (Zhao et al., 2019b). Together, all these data indicate that maintenance of cell wall integrity is critical for salt tolerance in plants. However, the molecular mechanisms underlying the regulation of cell wall biosynthesis under salt stress are still largely unknown.

Importin ɑ, also known as karyopherin αs (KPNAs), act as adaptors to form import complexes with importin β and cargo proteins, and drive the nuclear localization signal (NLS)-containing cargo proteins to the nucleus. In this process, hydrolysis of GTP by GTPase Ran provides the energy for the transport of import complex, and the asymmetric distribution of Ran in its GTP and GDP-bound states across the nuclear envelope determines the formation and disassembly of the import complex (Quimby & Dasso, 2003; Goldfarb et al., 2004). Beyond their typical role as adaptors in import complex, importin ɑ proteins also exhibit unexpected functions in eukaryotic organisms (Miyamoto et al., 2016; Oka & Yoneda, 2018). Several studies have reported the involvement of importin ɑ in spindle assembly and nuclear envelope formation in *Xenopus laevis* (Gruss et al., 2001; Nachury et al., 2001; Ems-McClung et al., 2004; Hachet et al., 2004; Wilbur & Heald, 2013). A study showed that NUP-6, an importin ɑ in *Neurospora*, is required for heterochromatin targeting, but not for nuclear transport of the factors that catalyze H3K9 methylation, suggesting a role of importin ɑ in delivering chromatin modifiers to the final destination beyond NLS-mediated nuclear transport (Klocko et al., 2015). Although the versatile roles of importin ɑ have been unveiled in eukaryotic cells, only a few studies have reported their functions in plants. There are nine importin α genes in Arabidopsis, and the functions of most of these genes are still unclear. MOS6 (or IMPα3) is critical for plant innate immunity (Palma et al., 2005). Several importin α isoforms can interact with *Agrobacterium tumefaciens* virulence proteins VirD2 and VirE2, but only IMPA4 was documented to be involved in the transport of T-DNA into the nucleus (Bhattacharjee et al., 2008). So far, the roles of importin α in abiotic stress response are still unknown. As another core member of import complex, importin β, has been reported to participate in abiotic stress responses in Arabidopsis. As a homolog of human importin β1, *AtKPNB1* is required for the regulation of ABA signaling pathway and drought stress response in Arabidopsis (Luo et al., 2013). SAD2 is another importin β-domain family protein involving in the nuclear import of an R2R3-type transcription repressor MYB4 in response to UV-B radiation (Zhao et al., 2007). The ABA hypersensitive phenotype of *sad2* mutant also reveals its role in ABA response (Verslues et al., 2006).

Agenet domain, a plant-specific homolog of Tudor domain, belongs to the Royal family domains. This family also includes Chromo (chromatin-binding), PWWP (Pro-Trp-Trp-Pro), and MBT (malignant brain tumour) domains (Maurer-Stroh et al., 2003). In Arabidopsis, there are 71 Agenet/Tudor-like domains in 32 different proteins (Liu & Min, 2016). SHH1 is one of the Agenet/Tudor-like proteins participating in RNA-directed DNA methylation pathway. The tandem Tudor-like repeats in the SAWADEE domain of SHH1 is required for the recognition of H3K9me2 and the recruitment of RNA polymerase IV to target loci (Law et al., 2011; Law et al., 2013). The tandem Agenet domains in AGDP1 specifically bind to H3K9me2 mark, which is required for DNA methylation in heterochromatin regions (Zhang et al., 2018; Zhao et al., 2019a). AIP1 is an Agenet domain-containing protein that functions as a linker between DNA replication, transcription, and chromatin remodeling during flower development (Brasil et al., 2015). The Agenet domain of EML1 was originally identified as a H3K4me3 reader (Zhao et al., 2018b), and later it was reported that EML1 and EML3 bind to peptides containing H3K36 and encompassed multiple modifications, including methylation and acetylation (Coursey et al., 2018). The EML1 and EML3 also recognize the H3K36 of viral genome and participate in viral chromatin regulation (Coursey et al., 2018), suggesting a role of Agenet domain proteins in defense response. So far the functions of Agenet domain proteins in abiotic stress response have rarely been reported. Here, we report a previously uncharacterized Agenet domain-containing protein SWO1 (SWOLLEN 1), which interacts with importin ɑ and functions in salt stress response via the regulation of cell wall remodeling. Our results suggest that SWO1 may function as a scaffold protein to deliver some nuclear-localized proteins to their targeted DNA regulatory regions.

## Results

### *SWO1* is required for root elongation under salt stress conditions

We collected a number of T-DNA insertion mutants that are disrupted in the genes encoding Agenet domain-containing proteins and analyzed their phenotypes under a variety of abiotic stresses. We found that a mutant (SAIL_236_A03), in which a T-DNA was inserted in the third intron of *AT4G17330*, showed a reduced root elongation when grown on ﻿MS solid medium supplemented with 100 mM NaCl (Fig. S1a, b). Another obvious phenotype observed in this mutant was the swollen root tip under high salinity, and thus we named this mutant as *swo1* (*swollen 1*), and the SAIL_236_A03 mutant allele was designated as *swo1–1*. To figure out whether the *swo1* mutant is also defective in the response to osmotic stress, we subjected the seedlings of the mutant to the MS medium supplemented with a high concentration (325 mM) of mannitol, which is often used to trigger osmotic stress. The result showed that the root elongation of the *swo1–1* mutant was not affected under osmotic stress (Fig. S1c, d).

**Fig. 1:**
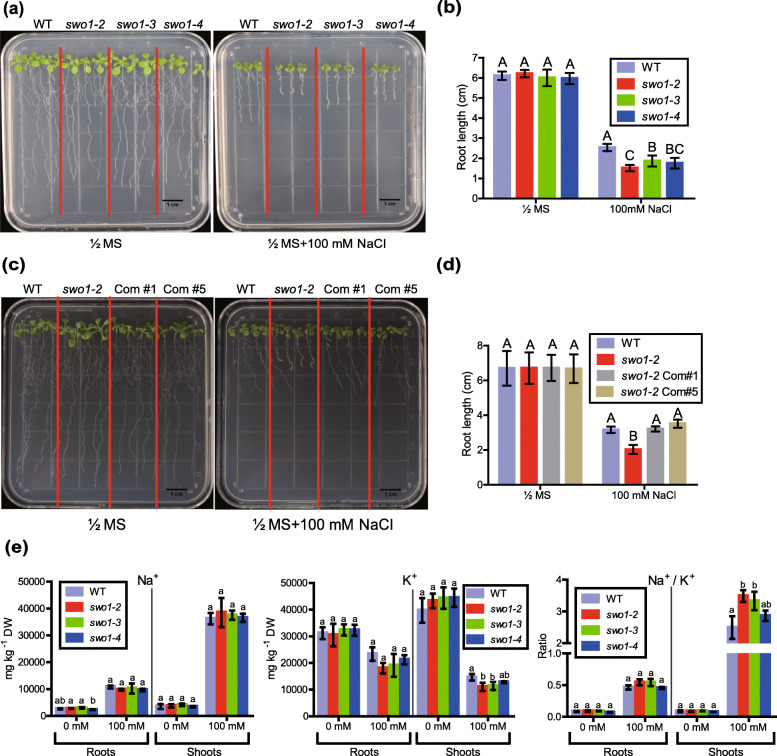
*SWO1* is required for root growth under salt stress. (**a**) Four-day-old seedlings of the wild type and the three *swo1* mutant alleles, *swo1–2*, *swo1–3*, and *swo1–4*, were transferred to Murashige and Skoog (MS) and MS + 100 mM NaCl media, respectively. The photographs were taken 7 days after transfer. (**b**) Quantification of the root lengths of the wild type and the *swo1* mutants after being transferred to MS and MS + 100 mM NaCl media for 7 days. The values indicate the means ± SD (*n* = 10). Different letters represent significant differences between different genotypes under the same treatment, *P* < 0.01 (one-way ANOVA). (**c**) Phenotypes of the wild type, *swo1–2*, and complementation plants after being transferred to MS media supplemented with or without 100 mM NaCl. The seedlings were photographed 7 days after growth. (**d**) Comparison of the root length of the wild type, *swo1–2*, and complementation plants after being transferred to MS and MS + 100 mM NaCl media for 7 days. Values are means ± SD (*n* = 8). Different letters represent significant differences between different genotypes under the same treatment, *P* < 0.01 (one-way ANOVA). (**e**) Analysis of Na^+^ and K^+^ concentrations and Na^+^/K^+^ ratio in the roots and shoots of the wild type and the *swo1* mutants after being transferred to agar-containing MS media with or without 100 mM NaCl for 10 days. Data are means ± SD (*n* = 3). Different letters indicate significant differences among the samples under the same treatment, *P* < 0.05 (one-way ANOVA). DW, dry weight

To further determine that the *SWO1* gene is required for salt tolerance in Arabidopsis, we tested the phenotypes of other three independent *swo1* T-DNA insertion mutant alleles, which were designated as *swo1–2*, *swo1–3*, and *swo1–4* (Fig. S2a). In these three mutant alleles, a T-DNA was inserted in the first, eighth, and thirteenth exon of the *SWO1* gene, respectively (Fig. S2a, b). Gene expression analysis showed that the transcript level of the *SWO1* gene was dramatically reduced in all these three mutant alleles (Fig. S2c, d). Similar to the *swo1–1*, all these three mutant alleles exhibited defective root growth under high salinity, including reduced root elongation and swollen root tips (Fig. 1a, b). We transformed the full-length genomic DNA of *SWO1* driven by its native promoter to the *swo1–1* and *swo1–2* mutant alleles. All complementation lines showed normal root elongation under high salinity compared with the wild type (Fig. 1c, d; Fig. S1e, f), demonstrating that *SWO1* is required for salt tolerance in Arabidopsis. Since in the *swo1–2*, *swo1–3*, and *swo1–4* mutant alleles, the T-DNAs are inserted in the exons of the *SWO1* gene, so mainly these three mutant alleles were used for further study.

To investigate whether the increased sensitivity of the *swo1* mutants to salt stress is due to abnormal ion homeostasis, we measured Na^+^ and K^+^ contents in both the roots and shoots of the wild type and *swo1* mutants. After being exposed to 100 mM NaCl, the wild type and *swo1* mutants did not show obvious difference in sodium concentration in both roots and shoots, but there was a slight decrease of potassium concentration in the shoots, but not in the roots of the *swo1* mutants (Fig. 1e). As a result, the Na^+^/K^+^ ratio was not affected in the roots but was increased in the shoots of the *swo1* mutants. These results suggested that the reduced root elongation of the *swo1* mutant under salt stress is not caused by the over-accumulation of Na^+^ in the roots.

### *swo1* mutation leads to abnormal root cell morphology and altered cell wall contents under salt stress

**Fig. 2:**
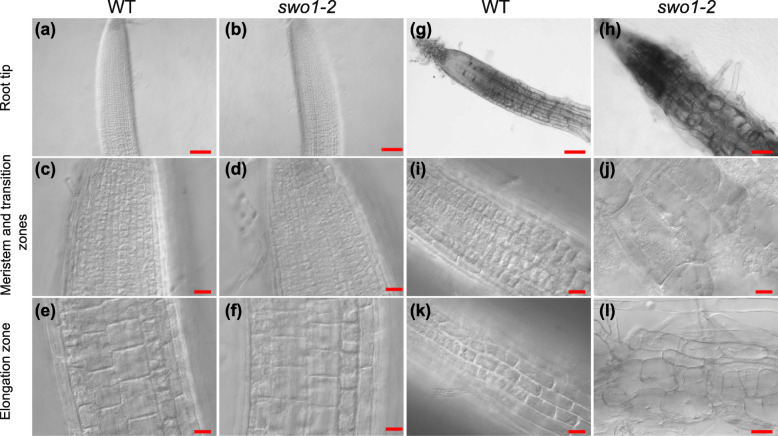
*swo1* mutation results in an abnormal root cell morphology under salt stress. (**a**)-(**f**). Morphology of the root tips of the wild type ((**a**), (**c**), and (**e**)) and *swo1–2* ((**b**), (**d**), and (**f**)) grown under normal conditions. (**a**) and (**b**), Scale bar = 100 μm. (**c**), (**d**), (**e**), and (**f**), Scale bar = 20 μm. (**g**)-(**l**). Morphology of the root tips of the wild type ((**g**), (**i**), and (**k**)) and *swo1–2* ((**h**), (**j**), and (**l**)) transferred to MS + 100 mM NaCl medium for 7 days. (**g**) and (**h**), Scale bar = 100 μm. (**i**), (**j**), (**k**), and (**l**), Scale bar = 20 μm. (**a**), (**b**), (**g**), and (**h**) are the morphological views of root tips. (**c**), (**d**), (**i**) and (**j**) show the root cells in the meristem and transition zones, and (**e**), (**f**), (**k**), and (**l**) show the elongation zone of the roots

The swollen root tip of the *swo1* mutant under salt stress drove us to closely observe the root tip cells using differential interference contrast (DIC) microscopy. The morphology of root cells was almost identical between the wild type and *swo1–2* under normal conditions (Fig. 2a-f). Under salt stress (100 mM NaCl), the wild type plants were able to maintain well-organized cell layers and defined cell shapes (Fig. 2g, i, k), whereas the *swo1* mutants displayed enlarged and disordered root cells in both meristem and elongation zones (Fig. 2h, j, l). The enlargement of root cells suggested that the *swo1* mutant loses the ability to tightly control cell expansion and growth under salt stress, which is likely caused by the disrupted cell wall integrity.

**Fig. 3:**
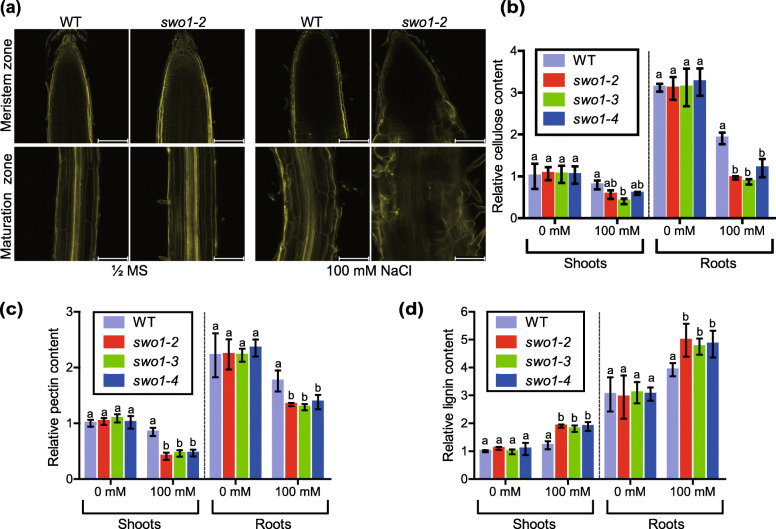
Cellulose and pectin contents are decreased in *swo1* mutants under high salinity. (**a**) Confocal microscopic images of calcofluor-stained root tips of the wild type and *swo1* mutant with or without NaCl treatment. The upper panel shows the images of the meristem zones and the lower panel shows the images of the maturation zones. Scale bar = 100 μm. (**b**) Quantitative analysis of cellulose content in the shoots and roots of the wild type and *swo1* mutants with or without NaCl treatment. The cellulose content in the shoot of the wild type without NaCl treatment was set as 1. Values are means ± SD (*n* = 3). Different letters represent significant differences between different genotypes under the same treatment, *P* < 0.05 (one-way ANOVA). (**c**) Relative amount of total pectin in the shoots and roots of the wild type and *swo1* mutants. The pectin content in the shoot of the wild type without NaCl treatment was set as 1. Values are means ± SD (*n* = 3). Different letters represent significant differences between different genotypes under the same treatment, *P* < 0.05 (one-way ANOVA). (**d**) Relative amount of lignin in the shoots and roots of the wild type and *swo1* mutants. The lignin content in the non-treated wild type shoots was set as 1. Values are means ± SD (*n* = 3). Different letters represent significant differences between different genotypes under the same treatment, *P* < 0.05 (one-way ANOVA)

Cell wall is an important factor that determines cell expansion during growth and development, as well as in response to environmental stresses (Shi et al., 2003; Xu et al., 2008). To determine whether the abnormal root cell morphology of the *swo1* mutant under salt stress is caused by the deficiency in cell wall integrity, we measured cell wall components, including cellulose, pectin, and lignin, in the wild type and the *swo1* mutants. To assess cellulose content, the roots of the seedlings grown on the MS solid medium supplemented with or without 100 mM NaCl for 6 days were stained with calcofluor, a special fluorescent dye that strongly binds to cellulose. Compared with the wild type, deposition of cellulose in the roots of the *swo1* mutant was markedly decreased after salt treatment (Fig. 3a). We then quantified the cellulose content in the wild type and *swo1* mutants using anthrone-sulfuric acid colorimetric assay. Under normal conditions, the cellulose content in the *swo1* mutants was comparable to that of the wild type. After salt treatment, however, the cellulose content was largely reduced in both the roots and shoots of the *swo1* mutants (Fig. 3b). Specifically, in the roots of the *swo1* mutants, the cellulose content was reduced by around 50% under salt stress. Salt stress also led to a dramatic decrease of pectin content in both the roots and shoots of the *swo1* mutants (Fig. 3c). In contrast, lignin content was significantly increased in the *swo1* mutants compared with the wild type in the presence of high salt, while the content of lignin was similar between the wild type and the *swo1* mutants under normal conditions (Fig. 3d). Together, these results suggest that the abnormal root cell morphology and root elongation of the *swo1* mutant under salt stress is largely caused by the compromised cellulose and pectin biosynthesis in the roots.

### Reactive oxygen species accumulation is responsible for salt stress-triggered root cell swelling in *swo1* mutant

**Fig. 4:**
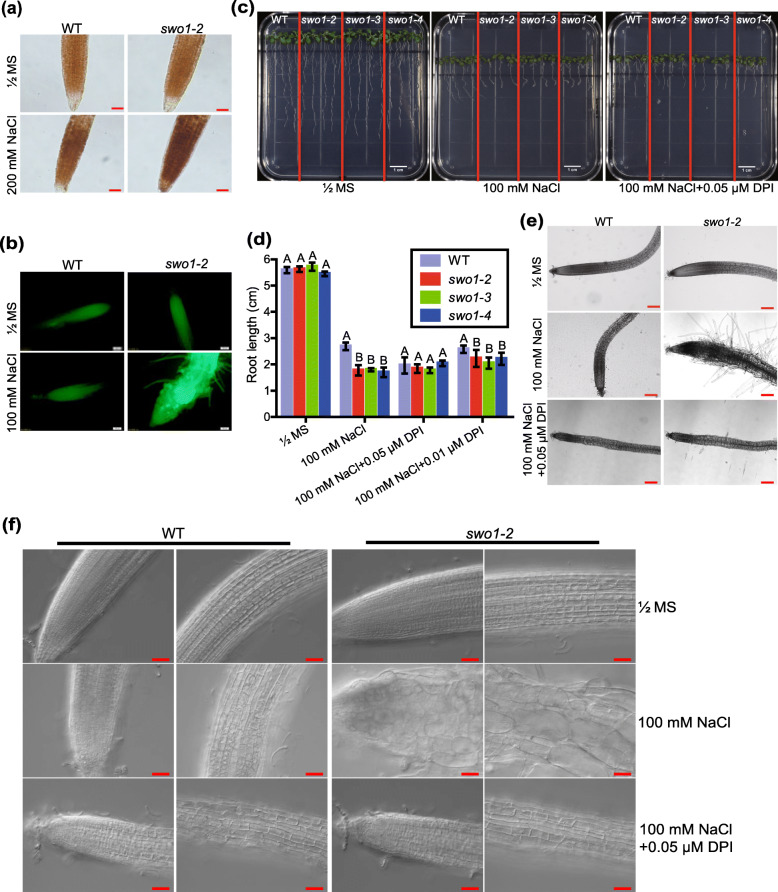
ROS accumulation is responsible for salt stress-triggered root cell swelling in *swo1* mutant. (**a**) DAB staining of H_2_O_2_ in the roots of the wild type and *swo1–2* mutant. The upper panel shows the staining of the roots grown under normal conditions, and the lower panel shows the staining of the roots treated with 200 mM NaCl for 6 h. Scale bar = 50 μm. (**b**) Analysis of ROS level by H_2_DCF-DA staining. Four-day-old seedlings of the wild type and *swo1–2* were transferred to MS media supplemented with or without 100 mM NaCl for 10 days. Roots were stained with H_2_DCF-DA and fluorescent signals were detected using an Olympus DP72 microscope. Scale bar = 100 μm. (**c**) Root phenotypes of the wild type and *swo1* mutants after being transferred to MS, MS + NaCl (100 mM), and MS + NaCl+DPI (0.05 μM) media for 7 days. (**d**) Quantification of the root lengths of the wild type and *swo1* mutants after being transferred to MS, MS + NaCl (100 mM), MS + NaCl+DPI (0.05 μM), and MS + NaCl+DPI (0.01 μM) media for 7 days. Data are means ± SD (*n* = 4). Different letters represent significant differences between different genotypes under the same treatment, *P* < 0.01 (one-way ANOVA). (**e**) Morphology of the root tips of the wild type and *swo1–2* after being transferred to MS, MS + NaCl (100 mM), and MS + NaCl+DPI (0.05 μM) media. The left panel shows the images of the wild type and the right panel shows the roots of *swo1–2*. Scale bar = 200 μm. (**f**) DIC images show the root cell morphology of the wild type and *swo1–2* grown on MS, MS + NaCl (100 mM), and MS + NaCl+DPI (0.05 μM) media for 7 days. Scale bar = 50 μm

Reactive oxygen species (ROS) have been proposed to be involved in the regulation of root growth and development, especially under stress conditions (Foreman et al., 2003; Tsukagoshi, 2016), and thus we examined ROS level in the roots of the *swo1* mutant under salt stress. DAB staining was applied to detect hydrogen peroxide content. Under normal growth conditions, there was no difference in H_2_O_2_ levels between the roots of the wild type and the *swo1* mutant (Fig. 4a, upper panel). However, after being suffered from high salinity, the *swo1* mutant showed more H_2_O_2_ accumulation in the roots than the wild type (Fig. 4a, lower panel). By using a fluorescent molecular probe H_2_DCF-DA, the pronounced accumulation of ROS in the root of the *swo1–2* mutant after salt stress was also observed (Fig. 4b).

To determine whether the over-accumulation of ROS is responsible for the salt-hypersensitive phenotype of the *swo1* mutant under salt stress, the four-day-old seedlings of the wild type and *swo1* mutants were transferred to NaCl media supplemented with NADPH-oxidase inhibitor diphenyleneiodonium chloride (DPI). After treatment of seedlings with 0.05 μM DPI, the root elongation of the wild type plants was inhibited, but the difference in root elongation between the wild type and *swo1* mutants under high salinity was largely abolished (Fig. 4c, d). On the salt medium supplemented with a reduced concentration of DPI (0.01 μM), the root elongation of the wild type plants was not obviously affected by DPI, but the root elongation rate of the *swo1* mutants was better than that on the salt medium without DPI (Fig. 4d). More importantly, application of DPI almost fully rescued the enlarged and disordered root cells of the *swo1* mutants under high salinity (Fig. 4e, f). Together, these results indicated that the over-accumulation of ROS is responsible for the reduced root elongation and swollen root cells of the *swo1* mutant under salt stress.

### *swo1* mutation affects the expression of ROS- and cell wall-related genes under salt stress

**Fig. 5:**
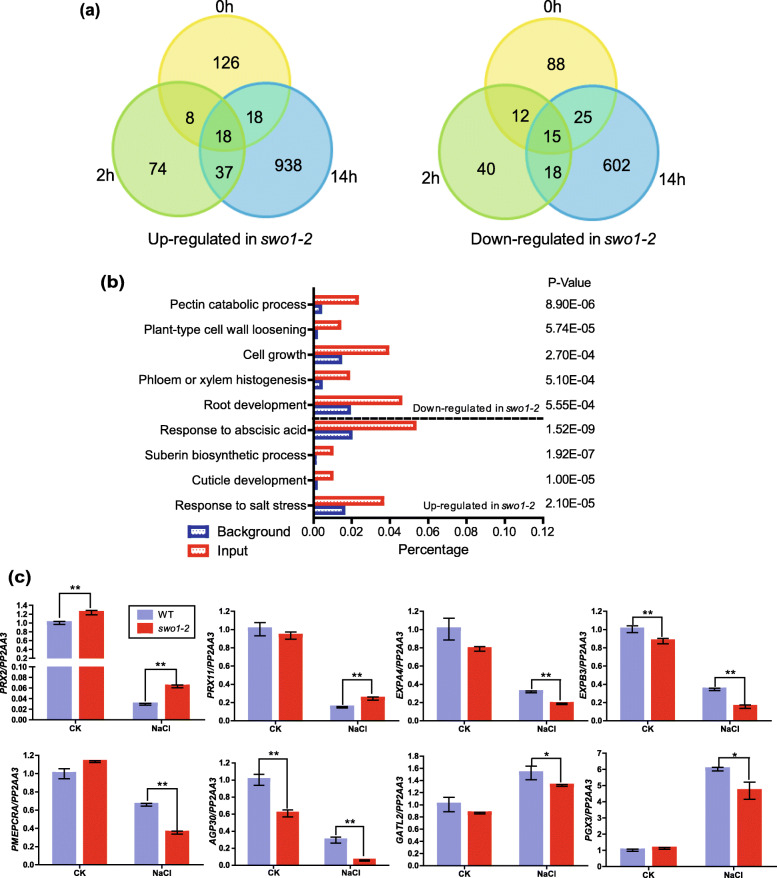
SWO1 regulates cell wall-related gene expression under saline conditions. (**a**) Venn diagrams show the numbers of up-regulated (left panel) and down-regulated (right panel) genes in *swo1–2* roots after salt treatment for 0, 2, and 14 h. (**b**) GO enrichment analysis of the differentially expressed genes (DEGs) in the *swo1–2* after salt treatment for 14 h. (**c**) qRT-PCR analysis of DEGs involved in ROS generation and regulation, cell wall modification and pectin biosynthesis in the *swo1–2* under salt treatment. *PP2AA3* was used as the internal control. Values are means ± SD (n = 3). **P < 0.01 and *P < 0.05 (Student’s *t* test)

To investigate the mechanisms underlying the reduced root elongation and enlarged root cells of the *swo1* mutant under high salinity, the roots of the wild type and *swo1–2* mutant after NaCl treatment for 0 h, 2 h, and 14 h were collected for RNA-seq analysis (Table S2). RNA-seq data revealed that 310 (170 up-regulated and 140 down-regulated) genes were differentially expressed in the *swo1–2* under normal conditions (fold change≥1.5, *P*-value<0.05). After salt treatment for 2 h, the number of the differentially expressed genes (DEGs) was still limited, with 137 genes up-regulated and 85 genes down-regulated in the *swo1–2* mutant (fold change≥1.5, P-value<0.05). After salt treatment for 14 h, however, totally 1671 DEGs (fold change≥1.5, P-value<0.05) were identified, and among of them 1011 genes were up-regulated and 660 genes were down-regulated in the *swo1–2* mutant (Fig. 5a).

The up- and down-regulated DEG sets after salt treatment for 14 h were subjected to gene ontology (GO) enrichment analysis. The up-regulated genes in the *swo1* mutant were significantly enriched in the categories “response to salt stress” (*p*-value = 2.10E-05), “response to ABA” (p-value = 1.52E-09), “cuticle development” (p-value = 1.00E-05), and “suberin biosynthetic process” (p-value = 1.92E-07) (Fig. 5b). Specifically, eight class III plant peroxidase family genes, which are supposed to be required for producing ROS and cell wall aromatic compounds oxidization, were up-regulated in the *swo1* mutant compared with the wild type under salt stress (Table S3). The down-regulated genes in the *swo1* mutant were classified into “root development” (p-value = 5.55E-04), “cell growth” (p-value = 2.70E-04), “pectin catabolic process” (p-value = 8.90E-06), “plant-type cell wall loosening” (*p*-value = 5.74E-05), and “phloem or xylem histogenesis” (*p*-value = 5.10E-04) (Fig. 5b). Among all the DEGs, 25 genes were identified to be involved in cell wall biogenesis and modification, and for most of them their expression was significantly reduced in the *swo1* mutant under salt stress (Table S3). These genes include pectin biosynthesis genes (*RGXT1*, *PGX3*, and *GATL2*), cell wall network-forming genes (*AGP30* and *AGP31*), cell wall modification genes (*PME* family members), and expansins (Table S3). Expansins are required for the regulation of cell expansion and growth via the modification of cell wall (Lee et al., 2001). Six members of the expansin family (*EXPA1*, *EXPA4*, *EXPA11*, *EXPA14*, *EXPA20*, and *EXPB3*) displayed decreased expression levels in the *swo1* mutant under salt stress (Table S3). To verify our RNA-seq data, several genes involved in ROS homeostasis, cell wall loosening and modification, as well as pectin biosynthesis, were selected for qPCR analysis, and the results indicated that their expression patterns are consistent with that generated in RNA-seq assay (Fig. 5c). In our RNA-seq data, we found that the expression of *MYB85*, one of the key transcription factors participating in lignin biosynthesis, was up-regulated in the *swo1* mutant root after salt treatment (Table S3), which is consistent with the increased lignin accumulation in the *swo1* mutant under salt stress.

### SWO1 directly binds to the genes involved in cell wall metabolism

**Fig. 6:**
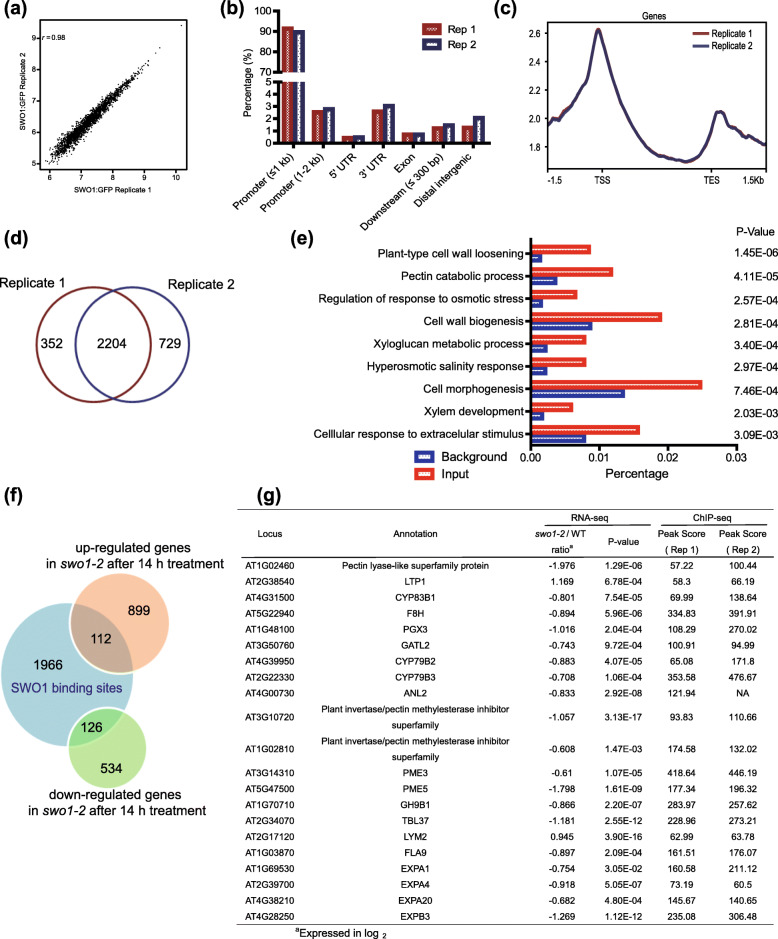
SWO1 binds to the promoters of cell wall metabolism-related genes. (**a**) Correlation analysis of SWO1 ChIP-seq data from two independent biological replicates. The X and Y axes represent read coverage normalized by total number of mapped reads in Log_2_ scale. Dot in each scatter plot shows the identified peaks in both replicates. (**b**) Genome-wide analysis of the binding preference of SWO1 in two ChIP-seq replicates. (**c**) Binding preference of SWO1 on gene bodies. TSS represents transcription start site and TES represents transcription end site of the nearest gene. (**d**) Analysis of the number of target genes that were identified in both biological replicates. (**e**) GO enrichment analysis of the SWO1-binding genes that were identified in both biological replicates. (**f**) Comparison of the genes generated from RNA-seq and ChIP-seq. Venn diagram shows the overlap of genes that are bound by SWO1 and are differentially expressed in the *swo1–2* after salt treatment for 14 h. (**g**) Listed are the cell wall-related genes that are bound by SWO1 and are differentially expressed in the *swo1–2* after salt treatment for 14 h

It has been reported that the Agenet/Tudor-like domain is able to bind to histones (Nielsen et al., 2002; Min et al., 2003; Zhang et al., 2018; Zhao et al., 2019a). To identify the genomic binding sites of SWO1, the genome-wide ChIP-seq assay was performed. Two independent biological replicates were conducted (Table S4). The two replicates exhibited a high identity based on correlation analysis (*r* = 0.98) (Fig. 6a). The majority of the binding events occurred in the promoter regions (Fig. 6b), and the summits of the most binding sites were identified near the transcription start sites (TSS) or transcription end sites (TES) (Fig. 6c). Totally 2236 significant peaks, representing 2204 genes were identified in both replicates (Fig. 6d). To determine the biological functions of the target genes of SWO1, we performed GO enrichment analysis for all of the 2204 genes. The result revealed a significant enrichment of the terms involved in “regulation of response to osmotic stress” (*p*-value = 2.57E-04), “hyperosmotic salinity response” (*p*-value = 2.97E-04), “cellular response to extracellular stimulus” (*p*-value = 3.09E-03), suggesting that *SWO1* is involved in the regulation of stress response. Importantly, the SWO1 binding sites were also enriched in the categories associated with plant cell wall metabolism, such as “xylem development” (*p*-value = 2.03E-03), “pectin catabolic process” (*p*-value = 4.11E-05), “cell wall biogenesis” (*p*-value = 2.81E-04), “xyloglucan metabolic process” (*p*-value = 3.40E-04), and “cell wall loosening” (p-value = 1.45E-06) (Fig. 6e), suggesting that SWO1 directly binds to the cell wall-related genes.

To determine whether the expression of the SWO1-binding genes was affected in the *swo1* mutant, we compared the data generated from ChIP-seq and RNA-seq assays. The result showed that 238 SWO1-binding genes were differentially expressed in the *swo1* mutant under high salinity, among of which 112 genes were up-regulated, and 126 genes were down-regulated (Fig. 6f). Among all the down-regulated genes, 19 are involved in plant cell wall metabolism (Fig. 6g, S3), including *F8H*, *EXPAs*, *GH9B1*, *PGX3*, and *PMEs*. *F8H* is required for the biogenesis of glucuronoxylan (Lee et al., 2009). Expansin genes (*EXPA1*, *EXPA4*, *EXPA20*, and *EXPB3*), as well as *GH9B1*, are involved in the regulation of plant growth and cell elongation (Shani et al., 2006). *PGX3* is required for the maintenance of cell wall integrity under salt stress (Zheng et al., 2019). Cell wall biosynthesis gene *PME3* contributes to the resistance of Arabidopsis to specific metal ion stresses (Weber et al., 2013) (Fig. 6g). Altogether, these results suggested that SWO1 regulates the expression of cell wall-related genes by directly binding to their promoters, and thus maintains root cell wall integrity under salt stress. For the ROS production-related genes that were differentially expressed in the *swo1* mutant, no significant binding peaks were identified, which suggests that SWO1 may not directly regulate the expression of these ROS production-related genes.

### The C-terminal domain of SWO1 is required for its nuclear localization

**Fig. 7:**
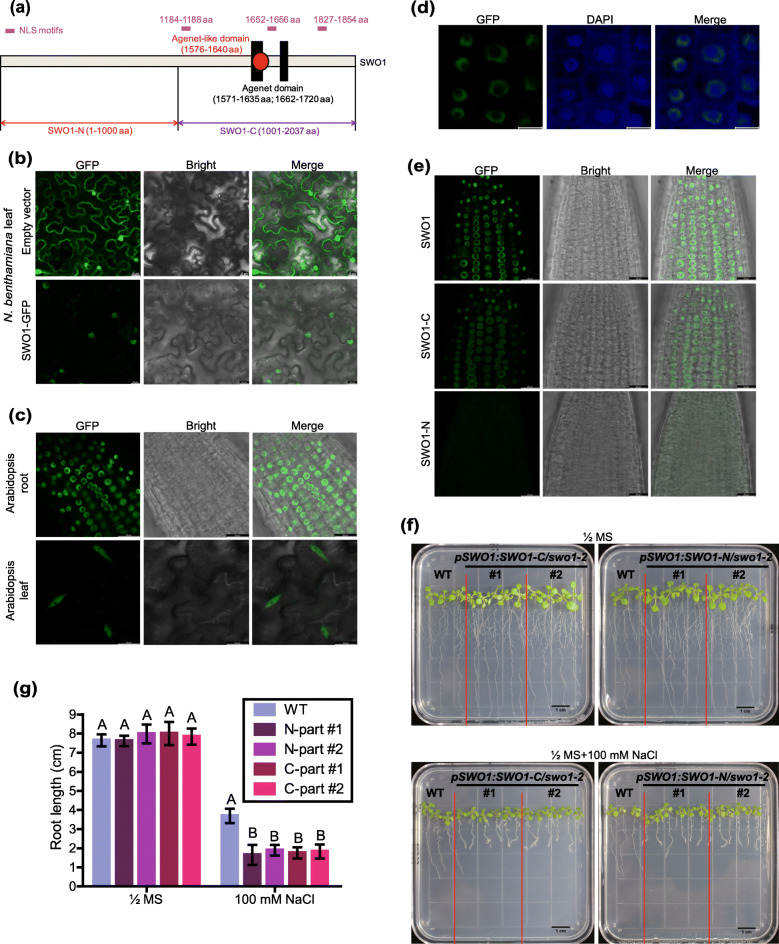
Both N- and C-termini are required for the function of SWO1 in the regulation of salt tolerance. (**a**) Schematic diagram shows the N-terminal and C-terminal domains of SWO1 protein. Black rectangles and red dot represent plant Agenet and Agenet-like domains, respectively, with the corresponding amino acid positions in the parentheses. The positions of the three NLS motifs are marked in purple. The fragments of SWO1-N and SWO1-C are also indicated in the diagram. (**b**) Subcellular localization of SWO1 in *N. benthamiana* leaves. The leaves expressing free GFP were used as control. Scale bar = 20 μm. (**c**) Subcellular localization of SWO1 in Arabidopsis roots (upper panel) and leaves (bottom panel). Scale bar = 20 μm. (**d**) Overlay between SWO1-GFP and DAPI (4′,6-diamidino-2-phenylindole) staining in Arabidopsis roots. Scale bar = 10 μm. (**e**) Subcellular localizations of the full-length and truncated SWO1 proteins in Arabidopsis roots were detected by confocal microscopy. Scale bar = 20 μm. (**f**) Four-day-old seedlings of the wild type and transgenic plants expressing the N-terminal or C-terminal domain of *SWO1* in *swo1–2* mutant background were transferred to MS and MS + NaCl (100 mM) media. The photographs were taken 7 days after transfer. (**g**) Quantification of the root lengths of the wild type and transgenic plants expressing truncated *SWO1* after being transferred to MS and MS + 100 mM NaCl media. Data are means ± SD (*n* = 6). Different letters represent significant differences between different genotypes under the same treatment, *P* < 0.01 (one-way ANOVA)

Based on the Pan-taxonomic Compara downloaded from Ensembl plants (https://plants.ensembl.org/index.html), we found that *SWO1* and its homologs only exist in plant species but not in animals and bacteria, indicating that *SWO1* is a plant-specific gene. We performed a BLASTP analysis of SWO1 protein against Arabidopsis database, and no paralogs of SWO1 were identified in Arabidopsis. The SWO1 is an extremely large protein (2037 aa), which consists of a tandem of conserved Agenet/Tudor-like domains and three predicted ﻿nuclear localization signal (NLS) motifs at the C-terminus, but no known motifs were identified at the N-terminus (Fig. 7a). Through the transient expression of *SWO1-GFP* driven by its native promoter in *Nicotiana benthamiana*, we found that SWO1 was localized exclusively in the nucleus, while as a control the free GFP was localized in both the cytosol and nucleus (Fig. 7b). We also checked the localization of SWO1 in Arabidopsis by using *pSWO1:SWO1-GFP* transgenic plants, which showed that SWO1 was localized in the nucleus in both leaves and roots (Fig. 7c, d).

To uncover which domain is required for the nuclear localization of SWO1, we generated stable transgenic Arabidopsis plants expressing the N-terminal or C-terminal part of *SWO1* in the *swo1–2* background. Similar to that of the full-length protein, the C-terminal domain that contains the NLS motifs was still localized in the nucleus (Fig. 7e), illustrating that the C-terminal domain is required for the nuclear localization of SWO1. However, in contrast to the full-length SWO1 that was distributed in the nucleoplasmic region close to the nuclear envelope, the C-terminal domain of SWO1 was relatively evenly distributed in the nucleoplasm (Fig. 7e, Fig. S4a), suggesting that the N-terminus is probably required for the attachment of SWO1 to nuclear envelope. Surprisingly, in the transgenic plants expressing the N-terminal domain, no fluorescence signals could be detected (Fig. 7e). To exclude the possibility that the low protein abundance of the N-terminal domain of SWO1 was caused by the abnormal expression of this truncated fragment, both semi- and quantitative reverse transcription-PCRs (RT-PCRs) were performed, and the results showed that the N-terminal domain of *SWO1* was transcribed normally in the transgenic plants (Fig. S4b, c). To understand the biological functions of the N-terminal and C-terminal domains of SWO1, the transgenic plants expressing the N-terminus and C-terminus of *SWO1* in the *swo1–2* mutant background were subjected to NaCl treatment, and neither of these transgenic plants could complement the reduced root elongation and swollen root tips of the *swo1–2* mutant under high salinity (Fig. 7f, g), suggesting that both the N-terminal and C-terminal domains are essential for the function of SWO1 protein. To test whether salt stress affects the subcellular localization of SWO1, we treated the *pSWO1:SWO1-GFP* complementation plants with 100 mM NaCl in a time-course manner, which showed that the localization of SWO1 was not obviously changed under salt stress (Fig. S5).

### SWO1 interacts with importins IMPA1 and IMPA2

**Fig. 8:**
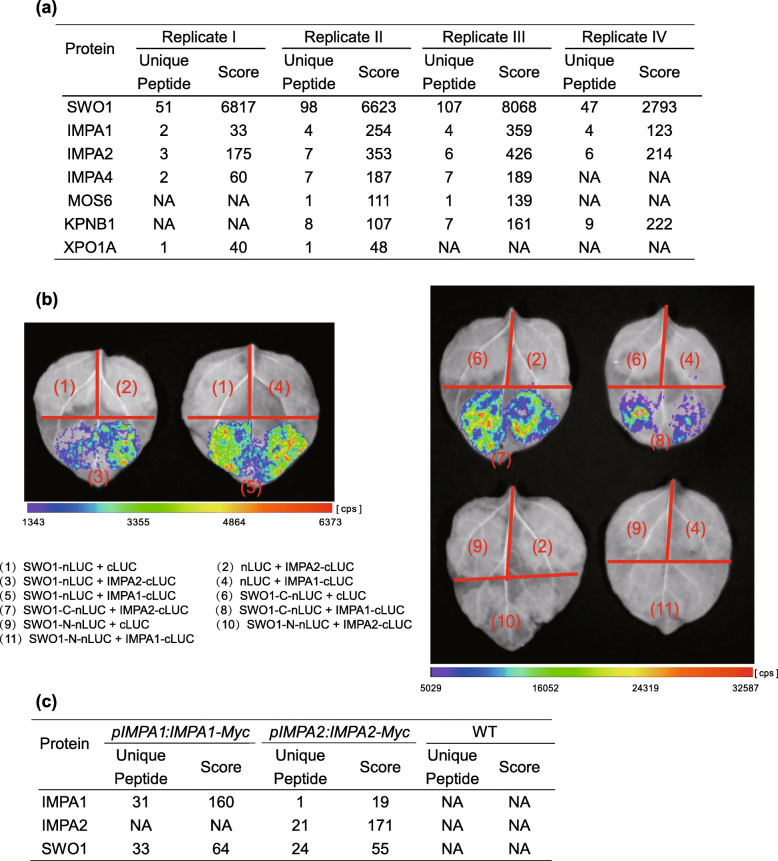
SWO1 interacts with importin ɑ IMPA1 and IMPA2. (**a**) Mass spectrometric analysis of SWO1-interacting proteins. The co-purified proteins, including IMPA1, IMPA2, IMPA4, MOS6, KPNB1, and XPO1A are listed. NA means that the corresponding protein was not identified in the IP-MS data. (**b**) Split luciferase complementation assay was performed in *N. Benthamiana* leaves. The combinations of the indicated plasmids were co-transformed to *N. Benthamiana* leaves through *Agrobacterium* infiltration. Luciferase activity was determined 48 h after infiltration. nLUC represents the N-terminal fragment of firefly luciferase; cLUC represents the C-terminal fragment of firefly luciferase. (**c**) Mass spectrometry analysis of the proteins co-purified with IMPA1 and IMPA2. The SWO1 that was co-purified with IMPA1 and IMPA2 is shown. NA means that the corresponding protein was not identified in the IP-MS data

To further understand the functions of SWO1 in salt tolerance, affinity purification assay was performed using the transgenic plants expressing *pSWO1:SWO1g-Myc*, and mass spectrometric (MS) analysis was conducted to identify co-purified proteins. Totally four independent experiments were performed, and the co-purified proteins were analyzed. Among these proteins, several members of importins, including IMPA1, IMPA2, IMPA4, MOS6, KPNB1, and XPO1A, were identified in two or more independent replicates (Fig. 8a). Since IMPA1 and IMPA2 were identified in all of the four independent replicates, their interactions with SWO1 were further examined by split luciferase (split-LUC) assay in *N. benthamiana*, which showed that SWO1 interacted with both IMPA1 and IMPA2. Truncation assay indicated that the C-terminal part of SWO1 is sufficient for the interactions (Fig. 8b). We also performed IP-MS analysis for the transgenic Arabidopsis plants expressing *pIMPA1:IMPA1-Myc* or *pIMPA2:IMPA2-Myc*, and SWO1 was co-purified in both samples (Fig. 8c). These results strongly indicated that SWO1 interacts with IMPA1 and IMPA2 and other importins.

Apart from importins, several nuclear pore complex (NPC) components, including the cytoplasmic member LOS4, the outer and inner ring members Nup96, RAE1, and Nup155, and the central FG factors Nup98, Nup54, and Nup35, were co-purified with SWO1. In addition, LINC4, SUN1, and WIT1 that are localized within the nuclear envelope were also identified as the co-purified proteins of SWO1 (Table S5). These results suggested that SWO1 may directly attach to nuclear envelope.

### Mutations of *IMPA* genes enhance the salt-hypersensitivity of *swo1* mutant

**Fig. 9:**
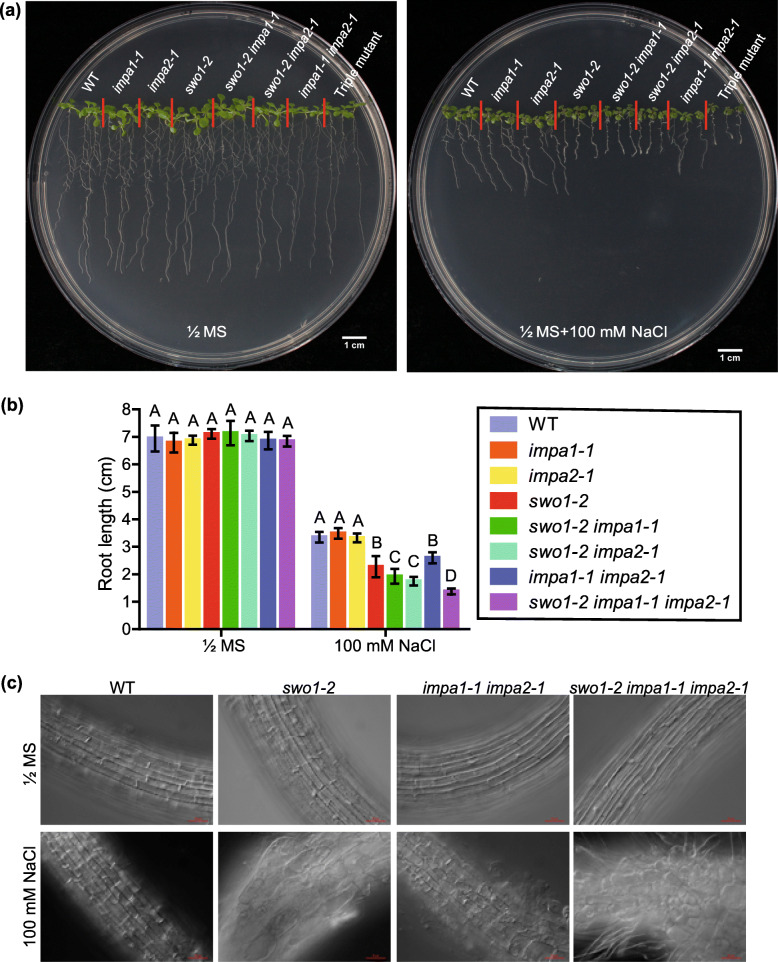
*IMPA1* and *IMPA2* are required for salt tolerance in Arabidopsis. (**a**) Phenotypes of the wild type, *swo1–2*, *impa1–1*, *impa2–1*, *swo1–2 impa1–1*, *swo1–2 impa2–1*, *impa1–1 impa2–1*, and *swo1–2 impa1–1 impa2–1* seedlings grown on MS and MS + 100 mM NaCl media. Photographs were taken 7 days after growth. (**b**) Root lengths of the wild type, *swo1–2*, *impa1–1*, *impa2–1*, *swo1–2 impa1–1*, *swo1–2 impa2–1*, *impa1–1 impa2–1*, and *swo1–2 impa1–1 impa2–1* seedlings grown on MS and MS + 100 mM NaCl media for 7 days. Data are means ± SD (*n* = 6). Different letters represent significant differences between different genotypes under the same treatment, *P* < 0.01 (one-way ANOVA). (**c**) Root cell morphology of the wild type, *swo1–2*, *impa1–1 impa2–1*, and *swo1–2 impa1–1 impa2–1* grown on MS and MS + NaCl (100 mM) media for 7 days. Scale bar = 50 μm

As SWO1 physically interacts with importins, we investigated the biological significance of these interactions and the roles of importin ɑ in salt tolerance. We obtained T-DNA insertion mutant lines of *IMPA1* and *IMPA2*, namely *impa1–1* and *impa2–1*, respectively. The double mutants *swo1–2 impa1–1*, *swo1–2 impa2–1*, and *impa1–1 impa2–1*, and the triple mutant *swo1–2 impa1–1 impa2–1* were generated by crossing and the homozygotes were identified. qRT-PCR results verified the absence of the transcripts of *IMPA1*, *IMPA2*, and *SWO1* in the corresponding mutants (Fig. S6). Both *impa1–1* and *impa2–1* single mutants showed similar phenotypes as the wild type under salt stress. However, the root elongation of the *impa1–1 impa2–1* double mutant was arrested under high salinity (Fig. 9a, b). Moreover, mutation in either the *IMPA1* or *IMPA2* gene enhanced the root growth inhibition of the *swo1–2* mutant under high salinity, and the triple mutant *swo1–2 impa1–1 impa2–1* showed even more severe root growth ﻿retardation and more serious root cell abnormality under salt stress (Fig. 9a, b, c). These results suggested that IMPA1 and IMPA2 are functionally redundant and work together with SWO1 in the regulation of root elongation under high salinity.

### SWO1 is not required for the NLS-mediated nuclear protein import

It is well-known that importins function in NLS-mediated nuclear protein import, and we tested whether *SWO1* acts as a component of importin complex. To this end, the GFP-NLS-CHS-NES(−)Rev construct, which expresses a cytosolic chalcone synthase (CHS) that is tagged with GFP-NLS and an inactivated nuclear export signal [NES(−)Rev], was transformed to the protoplasts of wild type, *impa1–1 impa2–1*, *swo1–2*, and *kpnb1* mutant. This construct has been used to elucidate the function of AtKPNB1 in the regulation of the shuttling of nuclear proteins (Luo et al., 2013). In the protoplasts derived from the wild type plants, the nuclear-localized GFP signal was detected in more than 50% of protoplasts (Fig. S7a). As expected, in the protoplasts of *kpnb1* mutant, less than 30% of protoplasts displayed GFP fluorescence signal in the nucleus (Fig. S7a). In the protoplasts generated from *impa1–1 impa2–1*, around 31% of protoplasts showed GFP fluorescence signal in the nucleus (Fig. S7a), indicating that mutations of both *IMPA1* and *IMPA2* affect the NLS-mediated import of nuclear proteins. However, in the protoplasts of the *swo1–2* mutant, the ratio of GFP signal in the nucleus was similar to that in the wild type, suggesting that *SWO1* mutation alone does not affect the NLS-mediated import of nuclear proteins (Fig. S7b).

Since IMPA1 and IMPA2 are required for NLS-mediated nuclear import (Fig. S7a), and SWO1 contains three NLS motifs, we were wondering whether SWO1 is the substrate of IMPA1 and IMPA2. We transformed *pSWO1:SWO1-GFP* to *swo1–2* and *impa1–1 impa2–1* mutants and compared the localization of SWO1 in these two transgenic plants. The results showed that the localization of SWO1 was indistinguishable in these two plant materials (Fig. S7c), suggesting that IMPA1 and IMPA2 are not required for the nuclear localization of SWO1. To test whether SWO1 functions in the recycling of IMPA1 and IMPA2 between the cytoplasm and nucleus, we also detected the subcellular localization of IMPA1 and IMPA2 in the *swo1* mutant, which showed that the localization of IMPA1 and IMPA2 was not changed in the *swo1–2* mutant (Fig. S7d), suggesting that SWO1 is not required for the regulation of the ﻿nucleocytoplasmic shuttling of IMPA1 and IMPA2.

## Discussion

In this study, we found that SWO1, a previously uncharacterized Agenet domain-containing protein, plays an important role in salt stress response via its associations with importin ɑ IMPA1 and IMPA2 in the nucleus. Both *swo1* and *impa1 impa2* mutants displayed enhanced root elongation inhibition under salt stress, and pyramiding these three mutations resulted in a more severe deficiency in root growth under high salinity. The *swo1* mutant exhibited root tip swelling under salt stress, which was largely caused by the disruption of cell wall integrity and increased accumulation of ROS. The role of SWO1 in the regulation of cell wall integrity was supported by the data showing that SWO1 binds to the promoters of many cell wall-related genes and regulates their expression under salt stress. Considering that SWO1 is a large protein that is physically associated with both importin ɑ and nuclear pore proteins and also binds to the promoters of the targeted genes, we speculate that SWO1 may act as an adaptor that links specific DNA regions to the NPC and thereby mediates the rapid and accurate delivery of the nuclear-imported proteins to their target genes (Fig. S8).

Cell wall is a specific structure that distinguishes plants from animals, and it fulfills diverse functions throughout the plant life. In addition to providing mechanical support and maintaining structural integrity, the wall is also the forefront to perceive a variety of environmental stimuli (Scheller & Ulvskov, 2010; Hamann, 2012). It has been well documented that cell wall integrity contributes to salt tolerance in plants. Under stress conditions, plants remodel the cell wall to adapt to unfavorable environments. Mutants that fail to maintain cell wall integrity usually exhibit a salt-hypersensitive phenotype, and typical phenotypes of cell wall-deficient mutants under salt stress are reduced root elongation and enlarged root cells (Shi et al., 2003; Zhu et al., 2010; Endler et al., 2015; Zhang et al., 2016; Zhao et al., 2018a; Zhao et al., 2019b). Here, mutation in the *SWO1* gene resulted in root cell swelling and reduced root elongation, and the cellulose and pectin contents were significantly reduced in the *swo1* mutant under salt stress, suggesting that SWO1 is required for the maintenance of cell wall integrity under high salinity. Notably, in most of the cell wall-deficient mutants, the root swelling under high salinity is usually restricted to the elongation zone, but in *swo1* mutant the root cell swelling extends to the meristem region. RNA-seq and ChIP-seq data indicated that SWO1 modulates cell wall remodeling by regulating the expression of many cell wall-associated genes, including pectin biosynthesis genes and cell wall modification genes, suggesting that the cell wall deficiency in the *swo1* mutant under salt stress is caused by the disrupted expression of cell wall-associated genes, but the molecular mechanism underlying the gene expression regulation by SWO1 needs to be further investigated.

In our study, we found that SWO1 directly interacted with importin ɑ IMPA1 and IMPA2, and importin β KPNB1. Combining with a previous study showing that SWO1 was co-purified with importin ɑ protein MOS6 (Roth et al., 2017), we can conclude that SWO1 is associated with the import complex. SWO1 harbors three NLS motifs, and whether these motifs are required for the interaction of SWO1 with import complex remains to be determined. The functional association of SWO1 with IMPA1 and IMPA2 was also genetically demonstrated in our study. Phenotypic analysis indicated that *impa1–1 impa2–1* double mutant exhibited reduced root elongation under salt stress, although the phenotype was weaker than the *swo1* mutant. Mutation of either *IMPA1* or *IMPA2* aggravated the short root phenotype of the *swo1* under salt stress conditions, and the triple mutant *swo1–2 impa1–1 impa2–1* showed even more retardation of root elongation and more severe root cell abnormality under high salinity, illustrating that SWO1 and IMPA1/IMPA2 function together to regulate salt tolerance in Arabidopsis. Since SWO1 also interacts with importin α members IMPA4 and MOS6, the relatively weak phenotype of the *impa1–1 impa2–1* double mutant compared with the *swo1* mutant could be explained by the functional redundancy of these importin α proteins. However, we cannot rule out the possibility that SWO1 may regulate salt tolerance via both importin α-dependent and -independent pathways.

Although the interaction of SWO1 with import complex is clear, the biological significance of their association is still elusive. One possibility is that SWO1 is a cargo of the import complex, because SWO1 contains three NLS motifs, but we did not detect the cytosolic retention of SWO1 in *impa1–1 impa2–1* mutant background, suggesting that the association of SWO1 with IMPA1 and IMPA2 is not critical for the nuclear import of SWO1. However, whether other importin α proteins function redundantly to regulate the transportation of SWO1 to the nucleus still needs to be investigated. Another possibility is that SWO1 functions as a member of the import complex to facilitate the import of other nuclear-localized proteins, but our results showed that *swo1* mutation did not affect the nuclear import of GFP driven by the NLS signal. Our data also showed that the nuclear localization of IMPA1 and IMPA2 was not affected in the *swo1* mutant. These results suggest that SWO1 does not participate in the process of the import of nuclear proteins. Since SWO1 is a large protein that interacts with several members of NPC and nuclear envelope, and is also capable of binding to the specific genomic regions, it is possible that SWO1 serves as a bridge between the nuclear envelope and chromatin, and the SWO1 protein may capture the imported nuclear proteins via its associations with the importin complex and then deliver the nuclear proteins to their target sites to regulate gene expression in response to salt stress.

The SWO1 protein contains Agenet domains that bind to histones, but it does not possess a transcriptional activity to directly promote gene expression, so it is likely that SWO1 acts as a harbor to deliver some unknown transcription factors or epigenetic regulators to the promoters of cell wall-associated genes. In our IP-MS data, we found a number of transcription factors, including zinc finger and bZIP families, that were co-purified with SWO1 (Table S6). Among these transcription factors, AtNFXL1 is required for plant growth under salt stress (Lisso et al., 2006), and OXS2 functions in salt tolerance (Jing et al., 2019). Roles of bZIP transcription factors in abiotic stress response have already been elucidated in previous studies. bZIP68 and VIP1 are involved in oxidative stress and mechanical stress response, respectively (Li et al., 2019; Tsugama et al., 2019), and bZIP59 regulates anthocyanin accumulation under salt stress in Arabidopsis (Van Oosten et al., 2013). In the future, the roles of these transcription factors in the regulation of cell wall-related genes expression need to be investigated. Agenet domain proteins have been documented as readers of histone modifications and function in epigenetic regulation (Nielsen et al., 2002; Min et al., 2003; Zhang et al., 2018; Zhao et al., 2019a). We searched for the candidate upstream regulators of the SWO1-targeted genes obtained from ChIP-seq using Plant Regulomics (Ran et al., 2020), and found a high enrichment of H2AK121ub in these genes. The histone modification H2AK121ub is written by H2A E3 ubiquitin ligase PRC1 and removed by ubiquitin-specific proteases UBP12 and UBP13 (Cao et al., 2005; Derkacheva et al., 2016). H2AK121ub is associated with a less accessible but still permissive chromatin at transcriptional regulation hotspots (Yin et al., 2021). H2AK121ub modification can repress gene expression via PRC2-mediated H3K27me3 deposition, while at the same time, it has an anti-repressive role possibly due to the recruitment of the H3K27-demethylase REF6 (Kralemann et al., 2020). In our IP-MS assay, UBP12 and UBP13 were co-purified with SWO1 (Table S5). Whether SWO1 transcriptionally regulates its target genes through binding and modifying H2AK121ub under salt stress remains to be studied. Since SWO1 also pulled down the chromatin remodeling protein CHR11 (Table S5) in the IP-MS assay, whether SWO1 is involved in chromatin remodeling and nucleosome patterning under salt stress needs further investigation. In brief, our study reveals a new component that is important for the regulation of cell wall integrity under salt stress and provides novel insights into the mechanisms that plants use to transduce environmental stimuli to the nucleus to control the expression of cell wall-associated genes under salt stress.

## Materials and methods

### Plant materials, growth conditions and genetic analysis

The Columbia ecotype of *Arabidopsis thaliana* was used in this study. The T-DNA insertion mutants *swo1–1* (SAIL_236_A03), *swo1–2* (SALK_201050C), *swo1–3* (SALK_206899C), *swo1–4* (SAIL_1284_D06), *impa1–1* (SALK_082616C), and *impa2–1* (SALK_149000C) were ordered from Arabidopsis Biological Resource Center (ABRC). The seeds were germinated and grown on half-strength Murashige & Skoog (MS) media for 7–10 days, and then the seedlings were transferred into soil and grown at 23 °C with a long-day light cycle of 16 h light/8 h dark.

Double and triple mutants were generated via hybridization, and the homozygous mutants were identified by PCR in the F_2_ population. Genomic DNAs isolated from 0.1 g of leaves were used for PCR. The primers used for PCR are listed in Table S1.

### Generation of transgenic plants

For complementation and subcellular localization analysis, we generated *SWO1pro:SWO1-GFP* construct, in which the expression of *SWO1-GFP* is driven by the native promoter of *SWO1* (2168 bp upstream region). The full-length genomic *SWO1* sequence was amplified using the wild type genomic DNA as a template. For the truncated SWO1 proteins fused with GFP, the promoter fragment was amplified from the wild type genomic DNA, and the coding sequences of the N (aa 1–1000) and C (aa 1001–2037) terminus were amplified using the wild type cDNA as a template. The fragments were cloned into pCAMBIA1300 vector with a GFP tag at the C terminus. These constructs were transformed into *swo1–2* plants. A similar procedure was applied for cloning the full-length genomic DNA of *SWO1* to pCAMBIA1305 vector with a 3 × Myc tag at the C terminus. The resulting *SWO1pro:SWO1g-Myc* construct was transformed to *swo1–1* mutant. The entire coding region of *IMPA1* or *IMPA2* together with 2 kb of the upstream fragments were amplified using the wild type genomic DNA as a template, and cloned into pCAMBIA1300-GFP and pCAMBIA1305–3 × Myc vectors, respectively. The primers used for constructs are listed in Table S1.

All clones were confirmed by sanger sequencing and the recombinant constructs were transformed into *A. tumefaciens* strain GV3101. Floral dip method (Clough & Bent, 1998) was used for transformation, and the transgenic plants were screened on the media containing 50 mg/L hygromycin.

### RNA-seq and gene expression analysis

For transcriptome analysis, seven-day-old wild type and *swo1–2* seedlings grown on vertical MS media were transferred to liquid MS media supplemented with 250 mM NaCl for 0, 2, and 14 h. Roots were collected and total RNAs were extracted using the RNeasy Plant Mini Kit (Qiagen) according to the manufacturer’s instructions. Sequencing was carried out by using Illumina NovaSeq 6000 platform. After processing of the raw data, clean reads were mapped to Arabidopsis genome sequences downloaded from TAIR 10 database. Differentially expressed genes (DEGs) were identified according to the published method (Robinson et al., 2010), with a criteria of fold change≥1.5 and *P*-value<0.05.

For gene expression analysis, total RNA was isolated from seven-day-old seedlings and 1 μg of the total RNA was reversely transcribed to cDNA using *TransScript*® One-Step gDNA Removal and cDNA Synthesis SuperMix (TransGen Biotech). Quantitative RT-PCR was carried out on a CFX96 real-time system (Bio-Rad) using SYBR Premix Ex-Taq (Takara) with the specific primers listed in Table S1.

### Abiotic stress treatment

For growth under stress conditions, Arabidopsis seeds were surface sterilized with 5% (v/v) NaClO containing 0.01% Triton-X-100 (v/v) for 10 min, and then washed 5 times before being sown on MS media. Four-day-old seedlings were transferred to vertical media supplemented with NaCl, mannitol, or chemicals under indicated concentrations and grown for the indicated times. The length of primary roots was measured using Image J software. One-way ANOVA or Student’s *t* test was used for statistical analysis.

### Quantification of Na^+^ and K^+^

Four-day-old seedlings of the wild type, *swo1–2*, *swo1–3*, and *swo1–4* were transferred to 100 mM NaCl media for 10 days. For comparison of Na^+^ and K^+^ accumulation, the roots and shoots of the treated plants were harvested, respectively, and rinsed five times with distilled water. All samples were fully dried at 65 °C for 4 days and weighed, followed by digestion with HNO_3_/HClO_4_ (85:15, v/v) at 135 °C for 4 h. The concentrations of Na^+^ and K^+^ were determined using Inductively Coupled Plasma Mass Spectrometry (ICP-MS, PerkinElmer NexION300D).

### Determination of cell wall components

To observe cellulose accumulation in roots after salt treatment, four-day-old seedlings were transferred to MS media with or without 100 mM NaCl. After growth for additional 6 days, roots were fixed in 2.5% glutaraldehyde in 0.2 M sodium phosphate buffer (PH 7.0) for 24 h at 4 °C, followed by staining with 0.2% calcofluor (Sigma-Aldrich). Images were taken using a confocal laser microscope (TCS SP8, Leica) at 405 nm.

To quantify the contents of cell wall polymers, the seedlings treated with NaCl for 14 days were harvested. Shoots and roots of the seedlings were detached and measured separately. For the quantification of cellulose and lignin contents, alcohol insoluble residue (AIR) was prepared and starch was removed according to Foster et al., (2010a). After sequential removal of lipids, proteins, and non-cellulosic polysaccharides, cellulose content was determined based on the quantification of glucose via the anthrone-sulfuric acid colorimetric assay (Scott & Melvin, 1953). Total lignin content was quantified via acetyl bromide soluble lignin (ABSL) assay (Foster et al., 2010b). To quantify the total pectin content, samples were fine powdered with liquid nitrogen and washed twice with 96% ethanol. The dried sediment was re-suspended with ddH_2_O and vacuum freeze dried. Pectin was finally dissolved and isolated in sulfuric acid overnight, and the content was determined colorimetrically according to a previous method (Blumenkr & Asboehan, 1973).

### Detection of reactive oxygen species

For H_2_O_2_ staining, seedlings were incubated in 1 mg mL^− 1^ 3, 3′-Diaminobenzidine (DAB) (Sigma) dissolved in 50 mM Tris-acetate buffer (PH 5.0) for 30 min in dark at room temperature. The seedlings stained with DAB were transferred to de-staining buffer (acetic acid: glycerol: ethanol = 1:1:3, v/v) and boiled for 10 min. Once chlorophyll was completely removed, the de-staining buffer was replaced with 95% ethanol (v/v), followed by visual score for color intensity. The 2′,7′- dichlorofluorescein diacetate (H_2_DCF-DA) staining assay for ROS estimation was performed as described previously (Foreman et al., 2003). In brief, seedling roots were incubated in 20 μM H_2_DCF-DA (Invitrogen) for 1 h at 4 °C and then washed with PBS buffer for 3 times. Fluorescent signals of ROS production were detected using microscope (DP72, Olympus).

### Microscopic observation

To observe root cell morphology of Arabidopsis under salt stress, four-day-old seedlings were transferred to 100 mM NaCl medium for 7 days. Roots were then excised and root tips were observed with differential interference contrast (DIC) under bright field using Axio Imager upright microscope (Zeiss). For the detection of subcellular localization, the roots of eight-day-old transgenic Arabidopsis, or the *Nicotiana Benthamiana* leaves that were transformed with plasmids for 2 days, were observed using confocal laser scanning microscopy (TCS SP8, Leica).

### Split-LUC complementation assay

The open reading frames of *SWO1*, *IMPA1*, and *IMPA2* were amplified from cDNA of the wild type Arabidopsis plants and cloned into pCAMBIA1300-nLUC and pCAMBIA1300-cLUC, respectively. To generate the truncated SWO1 proteins fused with LUC, the corresponding CDSs were cloned into the LUC vector to form SWO1-N (aa 1–1000) and SWO1-C (aa 1001–2038) constructs. The recombinant plasmids were transformed into *Agrobacterium* stain GV3101. After cultivation with 220 rpm at 28 °C overnight, *Agrobacteria* were harvested and re-suspended with infiltration buffer (10 mM MgCl_2_, 10 mM MES pH 5.6, and 100 μM acetosyringone) to a final concentration of OD_600_ = 0.5. Corresponding combinations of plasmids were infiltrated into *Nicotiana benthamiana* leaves after 2 h incubation at room temperature in dark. Followed by 48 h growth in chamber, tobacco leaves were sprayed with luciferin, and signals were detected using a CCD camera (PoLoN 1300B).

### Immunoprecipitation and mass spectrometry (IP-MS)

Total protein extraction and IP were performed as described previously (Law et al., 2010). In brief, epitope-tagged transgenic seedlings were powdered and suspended in lysis buffer (50 mM Tris pH 7.6, 150 mM NaCl, 5 mM MgCl_2_, 10% glycerol, 0.1% NP-40, 0.5 mM DTT, 1 mM PMSF, and protease inhibitor cocktail). Samples were completely homogenized, followed by centrifugation at 4 °C. Supernatants were incubated with pre-equilibrated anti-Myc agarose beads (Sigma). After incubation at 4 °C with rotation for 2 h, the beads were washed for 6 times with 5 min each time using lysis buffer, and 3 times with PBS buffer. The agarose beads were finally re-suspended in PBS buffer and used for mass spectrometry according to a previous publication (Wang et al., 2015).

### Arabidopsis protoplast assay

Arabidopsis protoplasts were isolated from three- to four-week-old wild type, *swo1–2*, *impa1–1 impa2–1,* and *kpnb1* plants grown under short day (10 h light/14 h dark) conditions. Well-expanded leaves were stripped with abaxial epidermis and submerged into the enzyme solution (1.5% cellulose R10, 0.4% macrozyme R10, 0.4 M mannitol, 20 mM KCl, 20 mM MES, 10 mM CaCl_2_, and 1% BSA). After digestion for 1–2 h in dark using a shaker at 50 rpm, an equal volume of W5 solution (0.1% Glucose, 0.08% KCl, 0.9% NaCl, 1.84% CaCl_2_·2H_2_O, and 2 mM MES) was added and the solutions containing protoplasts were filtered with a 55 μm nylon mesh. Followed by washing with W5 solution, the protoplasts were re-suspended in W5 solution, and kept on ice for 30 min in dark. After centrifugation, the concentration of cells was adjusted to 1–2 × 10^5^ mL^− 1^ using MMg solution (0.4 M mannitol, 15 mM MgCl_2_, and 4 mM MES) for transformation. PEG-mediated transformation was performed according to a previous method (Yoo et al., 2007), and fluorescence signals were observed using confocal laser scanning microscopy (TCS SP8, Leica) after transformation for 16 h.

### ChIP-seq assay

Chromatin immunoprecipitation (ChIP) assay was performed according to a previously described protocol with some modifications (Saleh et al., 2008). 5 g of the fourteen-day-old wild type and *SWO1pro:SWO1-GFP* seedlings were collected for cross-linking with 1% formaldehyde in PBS buffer and fine powdered in liquid nitrogen. Sonicated chromatin was incubated with anti-GFP magnetic beads GFP-Trap-MA (Chromotek) overnight. Immunoprecipitated DNA was purified with the QIAquick purification kit (Qiagen) and sent for sequencing. The Illumina Hiseq X Ten system was used for sequencing.

For ChIP-seq analysis, the pair-end reads were processed by Trimimomatic to trim the adapter sequences (Bolger et al., 2014). After the removal of low quality bases and the filter of short reads, clean reads were retained and mapped to the *A. thaliana* genome (TAIR10) by Bowtie 2 (version 2.2.8) (Langmead & Salzberg, 2012). Enriched peaks were identified by MACS (version 1.4) (Zhang et al., 2008), and the target genes were defined as the range from 1 kb upstream of transcription start site (TSS) to transcription end site (TES). The target genes of each peak were annotated by annotatePeak function in ChIPseeker package (Yu et al., 2015). The visualization of the average read coverage over gene body and additional 1.5 kb up- and down-stream of the TSS and TES was conducted by deepTools (version 2.4.1) (Ramirez et al., 2016). The statistical test of the overlap between two gene lists was performed by GeneOverlap package (Shen & Sinai, 2013).

### Data availability

﻿All data generated or analysed during this study are included in this published article and its supplementary information files. The RNA-seq and ChIP-seq data have been deposited in the NCBI GEO under accession number: GSE176434.

## Supplementary Information

**Additional file 1 Figure S1** Mutation in *SWO1* gene results in salt-hypersensitivity. (a) Phenotypes of the wild type and *swo1–1* seedlings grown on MS and MS + 100 mM NaCl media. Bar = 1 cm. (b) Quantification of the root lengths of the wild type and *swo1–1* grown on MS and MS + 100 mM NaCl media. Data are means ± SD (*n* = 6); ﻿**represents significant differences between the wild type and *swo1–1*, *P* < 0.01 (Student’s *t* test). (c) Phenotypes of the wild type and *swo1–1* grown on MS and MS + 325 mM mannitol media. (d) Quantification of the root lengths of the wild type and *swo1–1* grown on MS and MS + 325 mM mannitol media. Data are means ± SD (n = 6). (e) Phenotypes of the wild type, *swo1–1*, and *SWO1* complementation lines grown on MS and MS + 100 mM NaCl media. (f) Comparison of the root length of the wild type, *swo1–1*, and complementation plants after being transferred to MS and MS + 100 mM NaCl media. Values are means ± SD (*n* = 8). Different letters represent significant differences between different genotypes under the same treatment, P < 0.01 (one-way ANOVA).

**Additional file 2 Figure S2** Characterization of *swo1* mutants. (a) Schematic diagram shows the positions of the T-DNA insertions in different *swo1* mutant alleles. Black rectangles and lines represent exons and introns, respectively. Arrows and red lines indicate the primers used for genotyping. (b) Genotyping of the *swo1* mutant alleles using specific primers. (c) Semi-quantitative RT-PCR analysis of the transcript level of *SWO1* in the wild type and *swo1* mutant alleles. *ACTIN2* was used as the internal control. (d) qRT-PCR analysis of the transcript level of *SWO1* in the wild type and different *swo1* mutant alleles. *ACTIN2* was used as the internal control. Values are means ± SD (*n* = 3).

**Additional file 3 Figure S3** SWO1 binds to genes involved in cell wall metabolism. IGV screenshots show the cell wall-associated genes that were bound by SWO1 in ChIP-seq assay and were differentially expressed in the *swo1–2* after salt treatment for 14 h based on RNA-seq data.

**Additional file 4 Figure S4** Both N- and C-termini are required for the localization and function of SWO1. (a) The left panel shows the localization of full-length and C-terminal of SWO1 protein in the transition zone of Arabidopsis roots. The right panel is the evaluation of fluorescence intensities across corresponding nuclei. The small white rounds in the left panel represent the zero position in the right panel. Scale bar = 20 μm. (b) RT-PCR analysis of the transcript levels of *SWO1* N-terminal and C-terminal products in the wild type, *swo1–2*, and transgenic plants expressing full-length (SWO1-F), C-terminus (SWO1-C), and N-terminus (SWO1-N) of *SWO1*. *ACTIN2* was used as the internal control. (c) qRT-PCR analysis of the transcript levels of *SWO1* N-terminal and C-terminal products in the wild type, *swo1–2*, and transgenic plants expressing full-length and truncated *SWO1*. *ACTIN2* was used as the internal control. Values are means ± SD (n = 3).

**Additional file 5 Figure S5** Salt treatment does not affect the nuclear localization of SWO1. Time-course analysis of the subcellular localization of SWO1 after salt treatment from 0 min to 24 h in Arabidopsis roots. Scale bar = 20 μm.

**Additional file 6 Figure S6** Characterization of higher-order mutants. qRT-PCR analysis of the transcript levels of *IMPA1*, *IMPA2*, and *SWO1* in the wild type, *swo1–2*, *impa1–1*, *impa2–1*, *swo1–2 impa1–1*, *swo1–2 impa2–1*, *impa1–1 impa2–1*, and *swo1–2 impa1–1 impa2–1* seedlings. *ACTIN2* was used as the internal control. Values are means ± SD (n = 3).

**Additional file 7 Figure S7** SWO1 is not required for the import of nuclear-localized proteins. (a) Arabidopsis protoplasts generated from the wild type, *impa1–1 impa2–1*, and *kpnb1* were transiently transformed with the GFP-NLS-CHS-NES(−)Rev construct. Percentages of protoplasts emitting fluorescence from the nucleus were analyzed 16 h after transformation. Numbers of protoplasts used for analysis were shown on the columns (number of protoplasts emitting fluorescence from the nucleus/total number of transformed protoplasts). (b) Arabidopsis protoplasts generated from the wild type and the *swo1–2* mutant were transiently transformed with the GFP-NLS-CHS-NES(−)Rev construct, and fluorescence was observed 16 h after transformation. Scale bar = 50 μm. (c) Subcellular localization of SWO1 in *swo1–2* and *impa1–1 impa2–1* mutants. Scale bar = 20 μm. (d) Subcellular localization of IMPA1 and IMPA2 in their own mutants or *swo1–2* mutant. Scale bar = 20 μm.

**Additional file 8 Figure S8** A proposed working model. SWO1 may function as a linker between NPC and specific chromatin regions. Under stress conditions, the imported importin-cargo complex can be captured by SWO1 in the nucleus, which facilitates the delivery of nuclear regulatory effectors to their target sites to regulate downstream gene expression.

**Additional file 9 Table S1** Primers in this study.

**Additional file 10 Table S2** RPKM values of genes in RNA-seq assay.

**Additional file 11 Table S3** Shown are the class III peroxidases, cell wall loosening, and cell wall biogenesis and modification-related genes that are differentially expressed in *swo1–2* after salt treatment for 14 h.

**Additional file 12 Table S4** Peak information in SWO1 ChIP-seq assay.

**Additional file 13 Table S5** NPC, NE components and the factors involved in epigenetic regulation were identified in SWO1 IP-MS.

**Additional file 14 Table S6** Transcription factors that were identified in SWO1 IP-MS.

## Data Availability

All data generated or analysed during this study are included in this published article and its supplementary information files.

## Abbreviations

MS: Murashige and Skoog
NLS: Nuclear localization signal
NES: Nuclear export signal
DEGs: Differentially expressed genes
GO: Gene ontology
ICP-MS: Inductively coupled plasma mass spectrometry
AIR: Alcohol insoluble residue
ABSL: Acetyl bromide soluble lignin
ROS: Reactive oxygen species
DAB: 3, 3′-Diaminobenzidine
H2DCF-DA: 2′,7′-dichlorofluorescein diacetate
DPI: Diphenyleneiodonium chloride
DIC: Differential interference contrast
ChIP: Chromatin immunoprecipitation
TSS: Transcription start site
TES: Transcription end site
RT-PCR: Reverse transcription-PCR
IP-MS: Immunoprecipitation and mass spectrometry
split-LUC: Split luciferase
NPC: Nuclear pore complex
NE: Nuclear envelope
CHS: Chalcone synthase
IGV: Integrative genomics viewer
