# Supplementary figures and images for: SWO1 modulates cell wall integrity under salt stress by interacting with importin ɑ in Arabidopsis
Source: Stress Biol. 2021 Sep 29;1(1):9. doi: 10.1007/s44154-021-00010-5 (PMC10442049; doi:10.1007/s44154-021-00010-5)

**Figure S1**

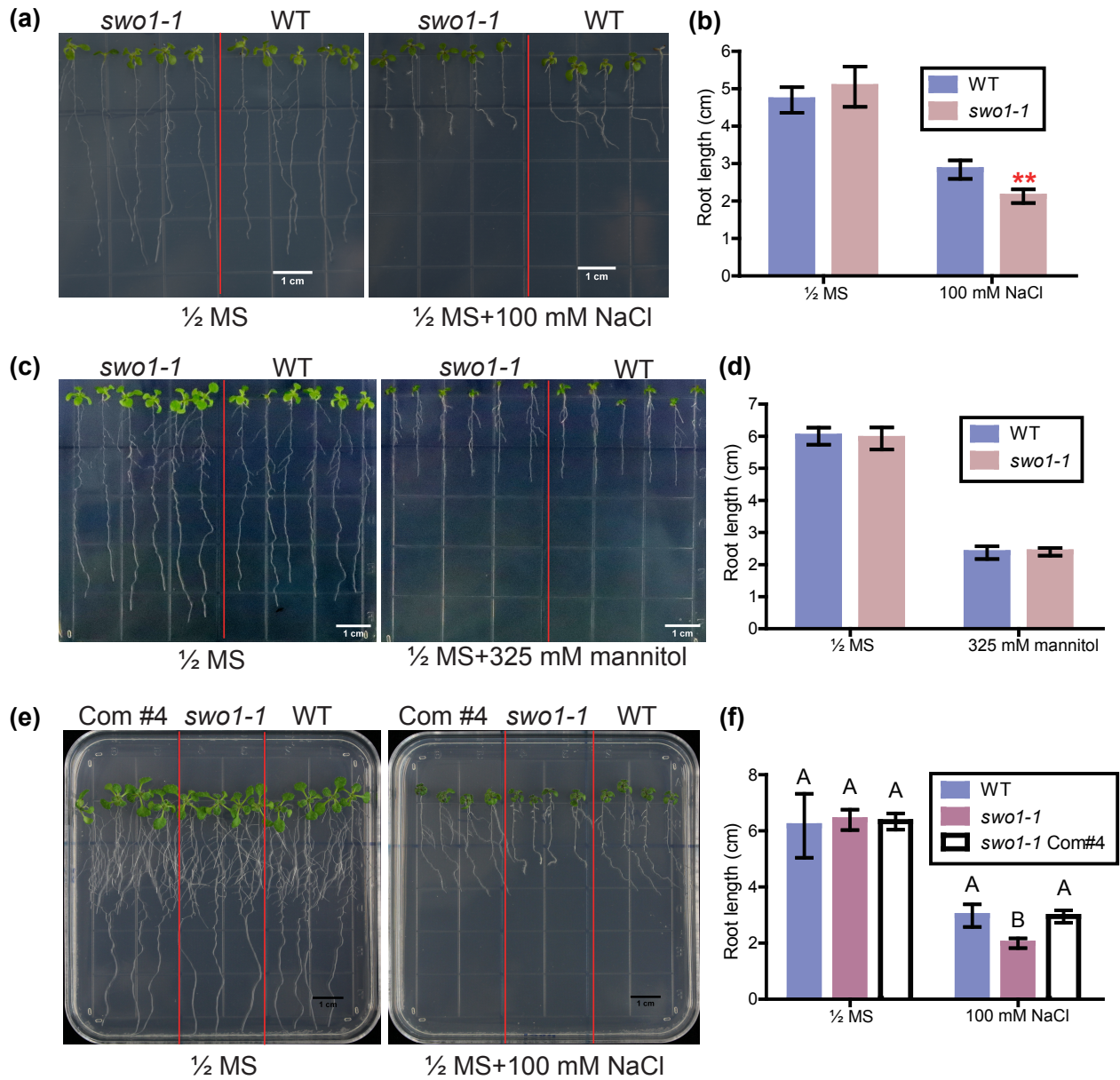

Supplement: Supplementary file 1 — Additional file 1 Figure S1 Mutation in SWO1 gene results in salt-hypersensitivity. (a) Phenotypes of the wild type and swo1–1 seedlings grown on MS and MS + 100 mM NaCl media. Bar = 1 cm. (b) Quantification of the root lengths of the wild type and swo1–1 grown on MS and MS + 100 mM NaCl media. Data are means ± SD (n = 6); ﻿**represents significant differences between the wild type and swo1–1, P < 0.01 (Student’s t test). (c) Phenotypes of the wild type and swo1–1 grown on MS and MS + 325 mM mannitol media. (d) Quantification of the root lengths of the wild type and swo1–1 grown on MS and MS + 325 mM mannitol media. Data are means ± SD (n = 6). (e) Phenotypes of the wild type, swo1–1, and SWO1 complementation lines grown on MS and MS + 100 mM NaCl media. (f) Comparison of the root length of the wild type, swo1–1, and complementation plants after being transferred to MS and MS + 100 mM NaCl media. Values are means ± SD (n = 8). Different letters represent significant differences between different genotypes under the same treatment, P < 0.01 (one-way ANOVA). [file 44154_2021_10_MOESM1_ESM.pdf]

Figure S2

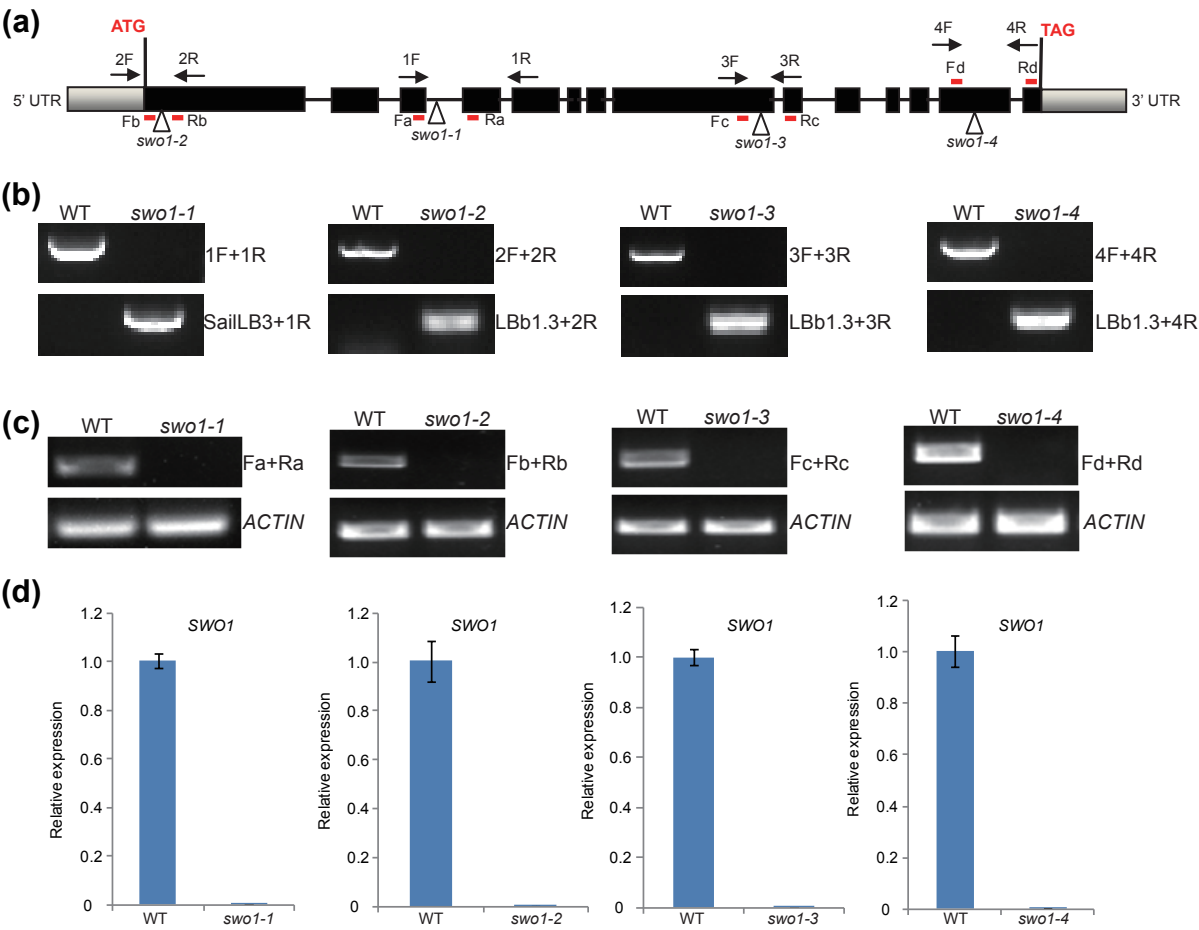

Supplement: Supplementary file 2 — Additional file 2 Figure S2 Characterization of swo1 mutants. (a) Schematic diagram shows the positions of the T-DNA insertions in different swo1 mutant alleles. Black rectangles and lines represent exons and introns, respectively. Arrows and red lines indicate the primers used for genotyping. (b) Genotyping of the swo1 mutant alleles using specific primers. (c) Semi-quantitative RT-PCR analysis of the transcript level of SWO1 in the wild type and swo1 mutant alleles. ACTIN2 was used as the internal control. (d) qRT-PCR analysis of the transcript level of SWO1 in the wild type and different swo1 mutant alleles. ACTIN2 was used as the internal control. Values are means ± SD (n = 3). [file 44154_2021_10_MOESM2_ESM.pdf]

Figure S3

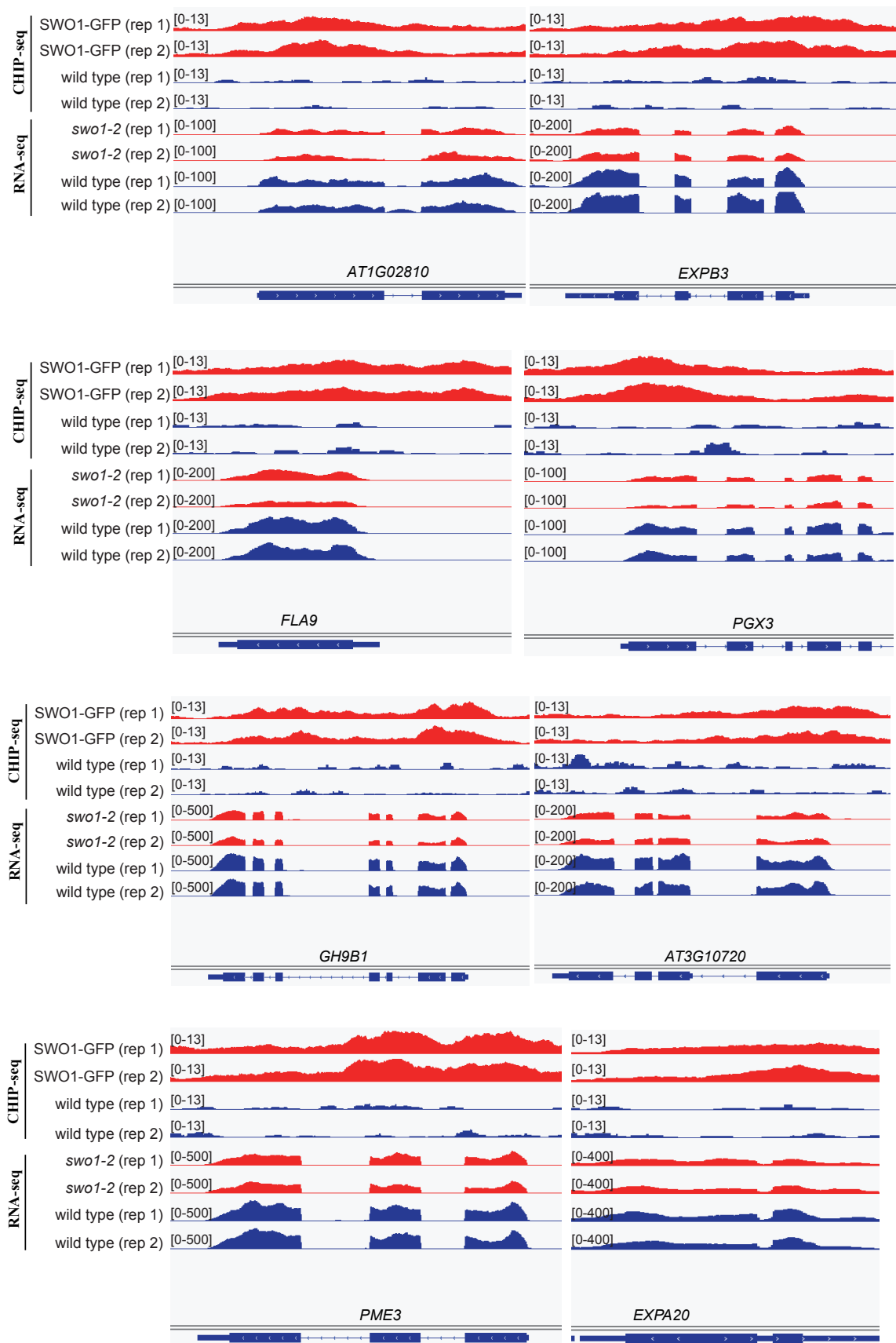

Supplement: Supplementary file 3 — Additional file 3 Figure S3 SWO1 binds to genes involved in cell wall metabolism. IGV screenshots show the cell wall-associated genes that were bound by SWO1 in ChIP-seq assay and were differentially expressed in the swo1–2 after salt treatment for 14 h based on RNA-seq data. [file 44154_2021_10_MOESM3_ESM.pdf]

**Figure S4**

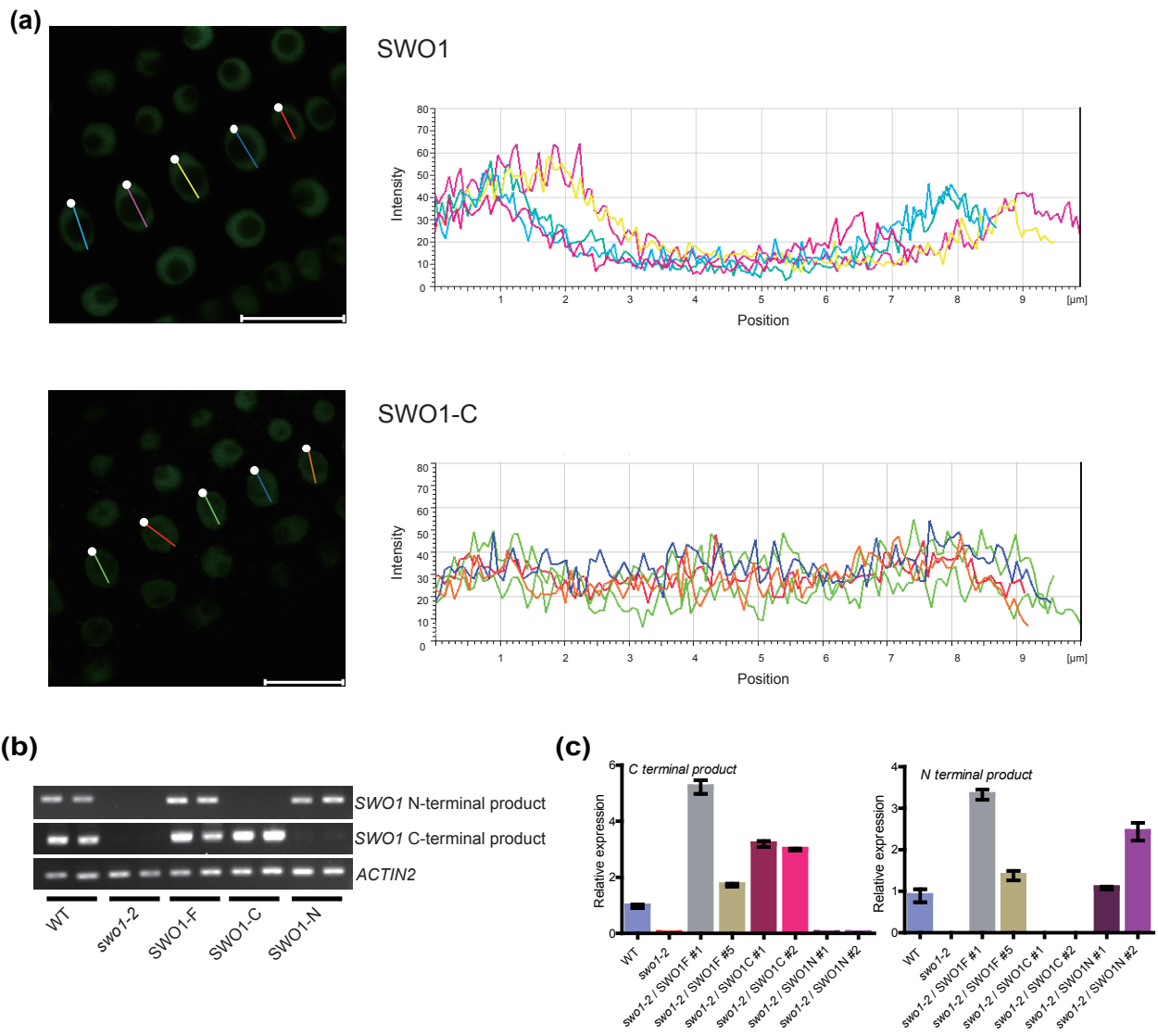

Supplement: Supplementary file 4 — Additional file 4 Figure S4 Both N- and C-termini are required for the localization and function of SWO1. (a) The left panel shows the localization of full-length and C-terminal of SWO1 protein in the transition zone of Arabidopsis roots. The right panel is the evaluation of fluorescence intensities across corresponding nuclei. The small white rounds in the left panel represent the zero position in the right panel. Scale bar = 20 μm. (b) RT-PCR analysis of the transcript levels of SWO1 N-terminal and C-terminal products in the wild type, swo1–2, and transgenic plants expressing full-length (SWO1-F), C-terminus (SWO1-C), and N-terminus (SWO1-N) of SWO1. ACTIN2 was used as the internal control. (c) qRT-PCR analysis of the transcript levels of SWO1 N-terminal and C-terminal products in the wild type, swo1–2, and transgenic plants expressing full-length and truncated SWO1. ACTIN2 was used as the internal control. Values are means ± SD (n = 3). [file 44154_2021_10_MOESM4_ESM.pdf]

**Figure S5**

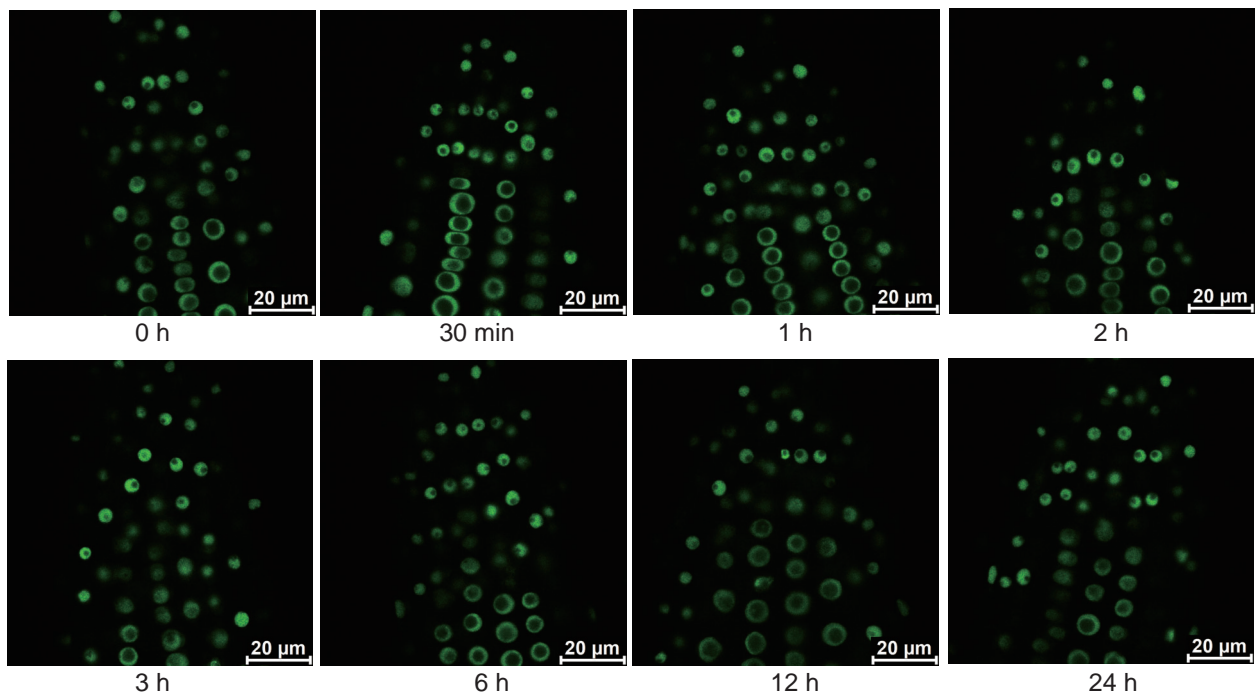

Supplement: Supplementary file 5 — Additional file 5 Figure S5 Salt treatment does not affect the nuclear localization of SWO1. Time-course analysis of the subcellular localization of SWO1 after salt treatment from 0 min to 24 h in Arabidopsis roots. Scale bar = 20 μm. [file 44154_2021_10_MOESM5_ESM.pdf]

Figure S6

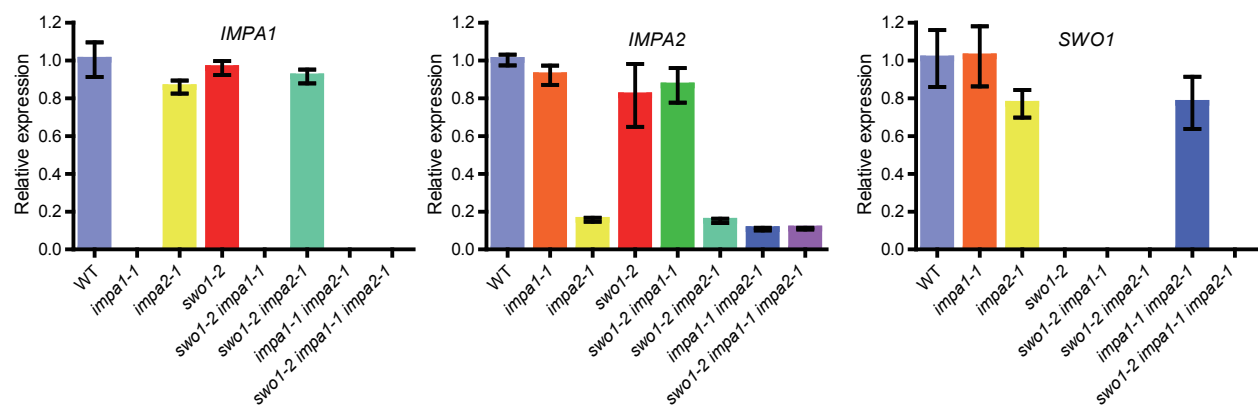

Supplement: Supplementary file 6 — Additional file 6 Figure S6 Characterization of higher-order mutants. qRT-PCR analysis of the transcript levels of IMPA1, IMPA2, and SWO1 in the wild type, swo1–2, impa1–1, impa2–1, swo1–2 impa1–1, swo1–2 impa2–1, impa1–1 impa2–1, and swo1–2 impa1–1 impa2–1 seedlings. ACTIN2 was used as the internal control. Values are means ± SD (n = 3). [file 44154_2021_10_MOESM6_ESM.pdf]

**Figure S7**

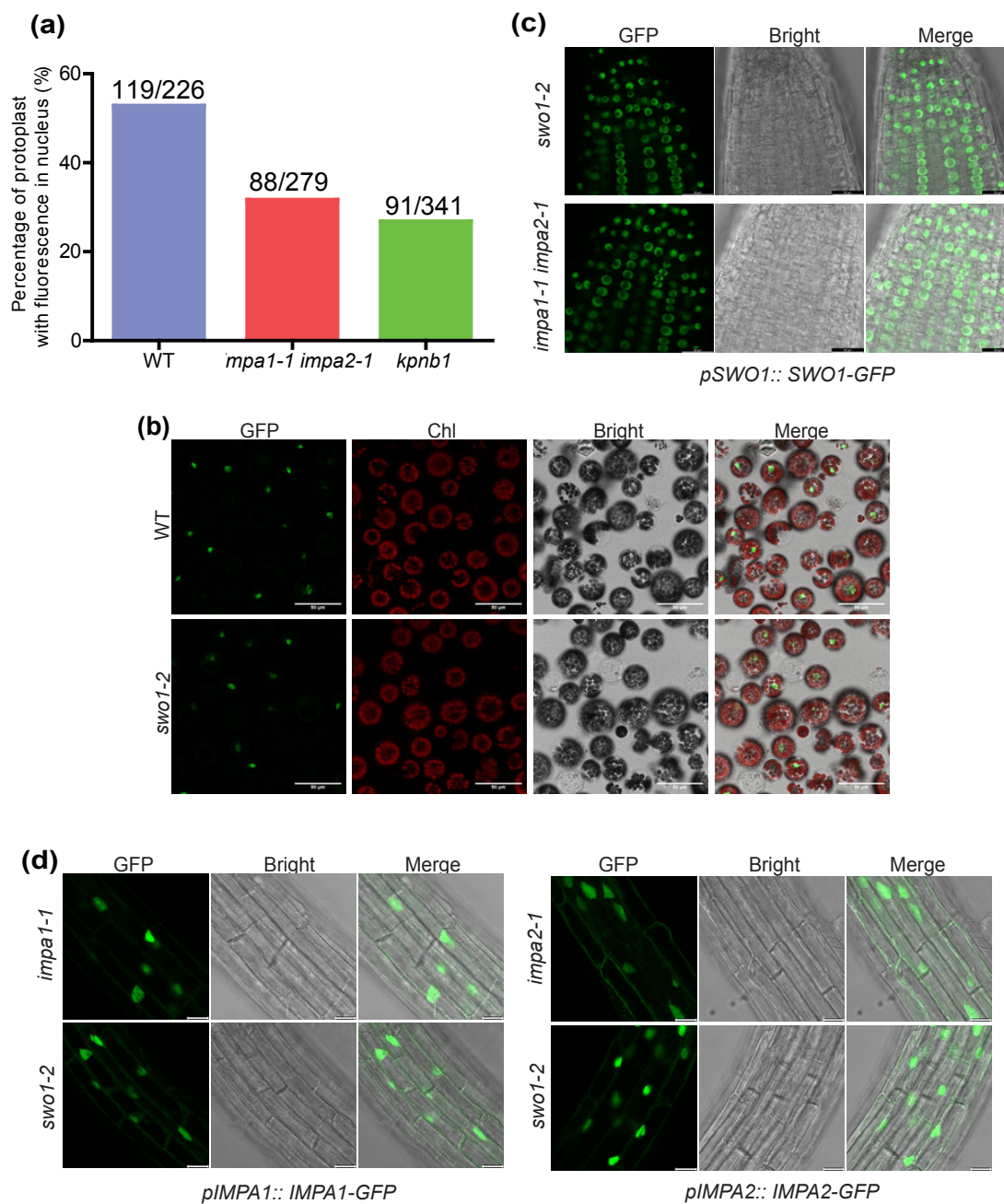

Supplement: Supplementary file 7 — Additional file 7 Figure S7 SWO1 is not required for the import of nuclear-localized proteins. (a) Arabidopsis protoplasts generated from the wild type, impa1–1 impa2–1, and kpnb1 were transiently transformed with the GFP-NLS-CHS-NES(−)Rev construct. Percentages of protoplasts emitting fluorescence from the nucleus were analyzed 16 h after transformation. Numbers of protoplasts used for analysis were shown on the columns (number of protoplasts emitting fluorescence from the nucleus/total number of transformed protoplasts). (b) Arabidopsis protoplasts generated from the wild type and the swo1–2 mutant were transiently transformed with the GFP-NLS-CHS-NES(−)Rev construct, and fluorescence was observed 16 h after transformation. Scale bar = 50 μm. (c) Subcellular localization of SWO1 in swo1–2 and impa1–1 impa2–1 mutants. Scale bar = 20 μm. (d) Subcellular localization of IMPA1 and IMPA2 in their own mutants or swo1–2 mutant. Scale bar = 20 μm. [file 44154_2021_10_MOESM7_ESM.pdf]

Figure S8

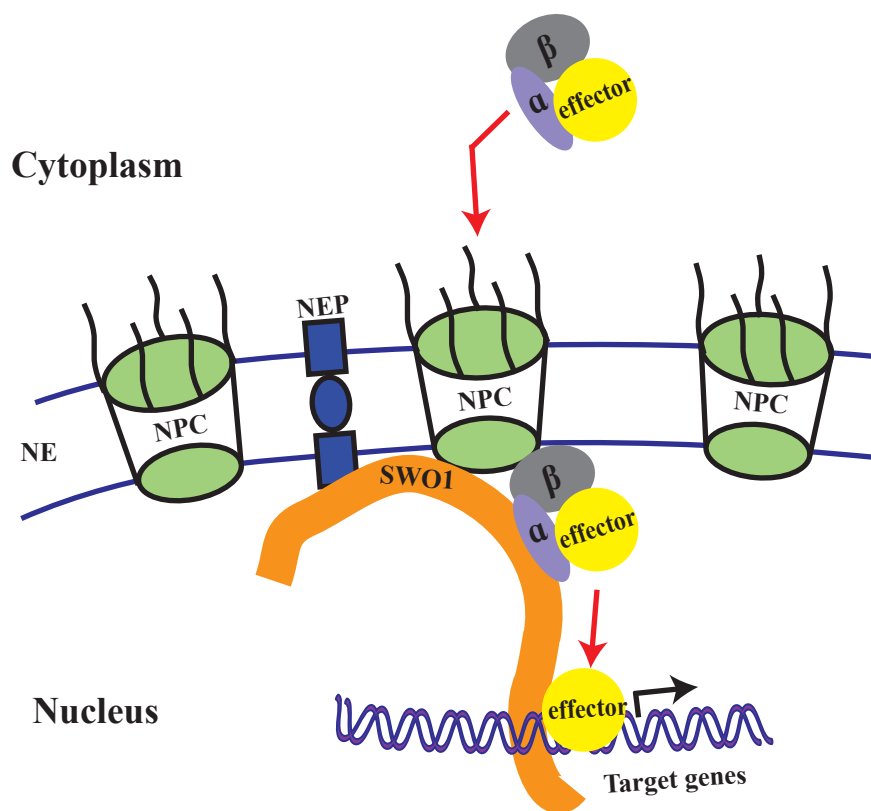

Supplement: Supplementary file 8 — Additional file 8 Figure S8 A proposed working model. SWO1 may function as a linker between NPC and specific chromatin regions. Under stress conditions, the imported importin-cargo complex can be captured by SWO1 in the nucleus, which facilitates the delivery of nuclear regulatory effectors to their target sites to regulate downstream gene expression. [file 44154_2021_10_MOESM8_ESM.pdf]
