# Supplementary material for: SWO1 modulates cell wall integrity under salt stress by interacting with importin ɑ in Arabidopsis
Source: Stress Biol. 2021 Sep 29;1(1):9. doi: 10.1007/s44154-021-00010-5 (PMC10442049; doi:10.1007/s44154-021-00010-5)
Supplement: Supplementary file 9 — Additional file 9 Table S1 Primers in this study. [file 44154_2021_10_MOESM9_ESM.docx]

| Name | Sequence (5’-3’) |
| --- | --- |
| Genotyping | |
| *swo1-1* LP | CTCCTTGCCTATTCTTGGGAG |
| *swo1-1* RP | GGTCGACGCAGAATACTTGAG |
| *swo1-2* LP | CCATGCTTTGCTCGAGATTAG |
| *swo1-2* RP | TACTTCGTCGGAATCTGTTGC |
| *swo1-3* LP | TTTTTACGCACCAGGAAAATG |
| *swo1-3* RP | TACAGCCCTGTAAAACGGTTG |
| *swo1-4* LP | GAATCTTCGAGGTCATCATCG |
| *swo1-4* RP | TGAATTGCAGGGTGATACTCC |
| *impa1-1* LP | GTGCTTTCATTCTTTGGATGC |
| *impa1-1* RP | TGTTTGTTAGAGCCCAAGCTG |
| *impa2-1* LP | CAATAGCCCAAAATGACTTGG |
| *impa2-1* RP | TGATTTGCCCTCTTGTCAATC |
| LBb1.3 | ATTTTGCCGATTTCGGAAC |
| SailLb3 | TAGCATCTGAATTTCATAACCAATCTCGATACAC |
| Transgenic constructs | |
| gSWO1-Myc-EcoRI-F | AGGAAACAGCTATGACCATGATTACGAATTCcttcctatctcaatcttcaccat |
| gSWO1-MR | TGAGTTTTCCAATAAGTGTATATTTTTAATAAAGAGCC |
| gSWO1-MF | tacacttattggaaaactcatga |
| gSWO1-Myc-HindIII-R | gctctctactagtccatggagatctaagcttctttttgctttgacttaggtgac |
| gSWO1-GFP-EcoRI-F | aggaaacagctatgaccatgattacgaattccttcctatctcaatcttcaccat |
| gSWO1-GFP-BamHI-R | ccatggtactagtgtcgactctagaggatccctttttgctttgacttaggtgac |
| cSWO1-N-GFP-MR | gttttgaaagtcactgtcatcataatccatgagtttattcctgaaatcagagac |
| cSWO1-N-GFP-MF | ttcaggaataaactcatggattatgatgacagtgactttc |
| cSWO1-N-GFP-BamHI-R | gtactagtgtcgactctagaggatccagtacgccatgatttctcccagc |
| cSWO1-C-GFP-MR | tgagcccttacaacacacatgagtttattcctgaaatcagagac |
| cSWO1-C-GFP-MF | ctgatttcaggaataaactcatgtgtgttgtaagggctcagaaa |
| gIMPA1-Myc-EcoRI-F | acagctatgaccatgattacgaattccttgggctatggcgtttatc |
| gIMPA1-Myc-HindIII-R | ctactagtccatggagatctaagcttgctgaagttgaatcctccgg |
| gIMPA2-Myc-EcoRI-F | acagctatgaccatgattacgaattcgtaagtactcatcttctgttac |
| gIMPA2-Myc-HindIII-R | ctactagtccatggagatctaagcttctggaagttgaatccacctg |
| Luc | |
| SWO1-nLuc-KpnI-F | agaacacgggggacgagctcggtaccatggattatgatgacagtgactttc |
| SWO1-nLuc-SalI-R | cgggacgcgtacgagatctggtcgacctttttgctttgacttaggtgac |
| SWO1-cLuc-KpnI-F | tctcgtacgcgtcccggggcggtaccatggattatgatgacagtgactttc |
| SWO1-cLuc-SalI-R | tacgaacgaaagctctgcaggtcgacctactttttgctttgacttaggt |
| SWO1-N-nLuc-KpnI-F | agaacacgggggacgagctcggtaccatggattatgatgacagtgactttc |
| SWO1-N-nLuc-SalI-R | cgggacgcgtacgagatctggtcgacagtacgccatgatttctcccagc |
| SWO1-N-cLuc-KpnI-F | tctcgtacgcgtcccggggcggtaccatggattatgatgacagtgactttc |
| SWO1-N-cLuc-SalI-R | tacgaacgaaagctctgcaggtcgactcaagtacgccatgatttctccc |
| SWO1-C-nLuc-KpnI-F | agaacacgggggacgagctcggtaccatgtgtgttgtaagggctcagaaa |
| SWO1-C-nLuc-SalI-R | cgggacgcgtacgagatctggtcgacctttttgctttgacttaggtgact |
| SWO1-C-cLuc-KpnI-F | tctcgtacgcgtcccggggcggtaccatgtgtgttgtaagggctcagaaa |
| SWO1-C-cLuc-SalI-R | tacgaacgaaagctctgcaggtcgacctactttttgctttgacttaggtg |
| IMPA1-nLuc-KpnI-F | agaacacgggggacgagctcggtaccatgtcactgagacccaacgctaa |
| IMPA1-nLuc-SalI-R | cgggacgcgtacgagatctggtcgacgctgaagttgaatcctccggatg |
| IMPA1-cLuc-KpnI-F | tctcgtacgcgtcccggggcggtaccatgtcactgagacccaacgctaa |
| IMPA1-cLuc-SalI-R | tacgaacgaaagctctgcaggtcgactcagctgaagttgaatcctccg |
| IMPA2-nLuc-KpnI-F | agaacacgggggacgagctcggtaccatgtctttgagacctaacgcta |
| IMPA2-nLuc-SalI-R | cgggacgcgtacgagatctggtcgacctggaagttgaatccacctgg |
| IMPA2-cLuc-KpnI-F | tctcgtacgcgtcccggggcggtaccatgtctttgagacctaacgcta |
| IMPA2-cLuc-SalI-R | tacgaacgaaagctctgcaggtcgactcactggaagttgaatccacct |
| (q)RT-PCR | |
| qActin2-F | GAGAGATTCAGATGCCCAGAAGTC |
| qActin2-R | TGGATTCCAGCAGCTTCCA |
| q*swo1-1*-F | CTTGCCTATTCTTGGGAGTGAAG |
| q*swo1-1*-R | CGGGAGTCTCCTAGTACGATTG |
| q*swo1-2*-F | GTTTGGTCTGAGGCTACTTC |
| q*swo1-2*-R | GGCTCTACTGTTTCCATTGT |
| q*swo1-3*-RT-F | TTTGGTGGCTCTGTGAATAC |
| q*swo1-3*-R | CATATTTATCACCCTCCTGTCC |
| *swo1-3*-RT-R | CTGAACAACACGTAAGCTTTATC |
| q*swo1-4*-F | GTGTCCCTAAACCTGGAAAG |
| q*swo1-4*-R | AATTGATCTCACAGGCTTAACT |
| q*impa1-1*-F | CTCCGTCGATAAGAAGTTGGATAG |
| q*impa1-1*-R | GAGAAGCTTCCTGAACTGAGTAG |
| q*impa2-1*-F | CAGAGAATACAAAGGTGGTCAT |
| q*impa2-1*-R | CACCAGCAACATTCCCTAAT |
| ACT2-RT-F1 | CTAAGCTCTCAAGATCAAAGGC |
| ACT2-RT-F2 | AACATTGCAAAGAGTTTCAAGG |
| *swo1-1*-RT-F | CAGCAGCAGTCTGAGGAATTA |
| *swo1-1*-RT-R | GGGAAAGAATCAAGAGGAGTCT |
| *swo1-2*-RT-F | TCTCGTGGAAGTAGTGGTATAG |
| *swo1-2*-RT-R | CATTGGCTTTCTCTTCTGTTTG |
| *swo1-4*-RT-F | CCTTCTCTTGTGTGGAAAGATG |
| *swo1-4*-RT-R | GGGCACAATTGATCTCACAG |
| q*PP2AA3*-F | CATTTCACTCCTCTGGCTAAG |
| q*PP2AA3*-R | GCTCCGTCTTTAGCACATC |
| q*PRX2*-F | CTGATGTTCTTGCCTTGGT |
| q*PRX2*-R | CATCGTTCAATTTCGAGATGC |
| q*PRX*11-F | AAGGTCTCTCGGTTGAAGA |
| q*PRX*11-R | TCGGAAGTTGCGACATTG |
| q*EXPB3*-F | TCCGTAACCGTGGACTAAT |
| q*EXPB3*-R | CAGTTGATCCTTCGTTCACA |
| q*EXPA4*-F | ACCACGTGAACACTTTGACCT |
| q*EXPA4*-R | GGTGACCGTTGATTGTGAAC |
| q*PMEPCRA*-F | TCATTTCCAATCGCTTCCC |
| q*PMEPCRA*-R | CGGTCACCTTAAGTGTCTTG |
| q*AGP30*-F | ACAACAAGACCCTAGTGGCAG |
| q*AGP30*-R | CACACAAGTCTCACCACCGC |
| qGATL2-F | GCATTGGAGTGGGAAAGGGA |
| qGATL2-R | AACCCAAAGTGCATCAAGCG |
| q*PGX3*-F | ATCGATTGGAACTGGCTGCT |
| q*PGX3*-R | AATTGTGCACGCCTAAGCTC |
