# Supplementary material for: SWO1 modulates cell wall integrity under salt stress by interacting with importin ɑ in Arabidopsis
Source: Stress Biol. 2021 Sep 29;1(1):9. doi: 10.1007/s44154-021-00010-5 (PMC10442049; doi:10.1007/s44154-021-00010-5)
Supplement: Supplementary file 11 — Additional file 11 Table S3 Shown are the class III peroxidases, cell wall loosening, and cell wall biogenesis and modification-related genes that are differentially expressed in swo1–2 after salt treatment for 14 h. [file 44154_2021_10_MOESM11_ESM.pdf]

Table S3. Shown are the class III peroxidases, cell wall loosening, and cell wall biogenesis and modification-related genes that are differentially expressed in *swo1-2* after salt treatment for 14 h

Locus	Annotation	$\log_2$ (*swo1* / WT)	P-value
Class III peroxidases			
AT1G05250	Prx02	1.22	4.14E-02
AT1G14540	Prx04	0.713	4.70E-02
AT1G68850	Prx11	1.049	1.45E-05
AT2G35380	Prx20	0.736	1.91E-02
AT2G38380	Prx22	0.717	1.02E-06
AT4G26010	Prx44	1.06	6.67E-03
AT4G33420	Prx47	0.652	8.95E-03
AT5G06730	Prx54	0.864	3.37E-03
Cell wall loosening			
AT1G69530	EXPA1	-0.754	3.05E-02
AT2G39700	EXPA4	-0.918	5.05E-07
AT1G20190	EXPA11	-0.703	1.12E-03
AT5G56320	EXPA14	-1.028	2.29E-05
AT4G38210	EXPA20	-0.682	4.80E-04
AT4G28250	EXPB3	-1.269	1.12E-12
AT3G45970	EXLA1	0.778	6.17E-06
AT4G38400	EXLA2	1.128	1.62E-05
AT3G45960	EXLA3	0.737	3.68E-02
AT4G17030	EXLB1	1.245	5.60E-25
Cell wall biogenesis and modification			
AT1G11545	XTH8	-0.739	9.56E-04
AT5G62380	NAC101	-1.049	1.11E-02
AT5G22940	F8H	-0.894	5.96E-06
AT1G48100	PGX3	-1.016	2.04E-04
AT1G68560	XYL1	-0.74	7.34E-12
AT2G45890	ROPGEF4	-0.775	4.96E-02
AT1G70710	GH9B1	-0.866	2.20E-07
AT1G14080	FUT6	-1.075	4.33E-07
AT1G67330	AGM2	-0.901	2.39E-12
AT4G00730	ANL2	-0.833	2.92E-08
AT1G03870	FLA9	-0.897	2.09E-04
AT2G33790	AGP30	-2.343	1.85E-21
AT1G28290	AGP31	-1.134	3.58E-17
AT4G01770	RGXT1	-1.406	7.32E-08
AT3G50760	GATL2	-0.743	9.72E-04
AT3G14310	PME3	-0.61	1.07E-05
AT5G47500	PME5	-1.798	1.61E-09
AT1G11580	PMEPCRA	-1.013	9.72E-12
AT3G10720	Plant invertase/pectin methylesterase inhibitor superfamily	-1.057	3.13E-17
AT1G02810	Plant invertase/pectin methylesterase inhibitor superfamily	-0.608	1.47E-03
AT1G02460	Pectin lyase-like superfamily protein	-1.976	1.29E-06
AT3G54260	TBL36	-0.861	8.05E-05
AT2G34070	TBL37	-1.181	2.55E-12
AT2G42570	TBL39	-0.632	4.50E-08
AT4G22680	MYB85	1.011	9.01E-07
