# Supplementary material for: SWO1 modulates cell wall integrity under salt stress by interacting with importin ɑ in Arabidopsis
Source: Stress Biol. 2021 Sep 29;1(1):9. doi: 10.1007/s44154-021-00010-5 (PMC10442049; doi:10.1007/s44154-021-00010-5)
Supplement: Supplementary file 13 — Additional file 13 Table S5 NPC, NE components and the factors involved in epigenetic regulation were identified in SWO1 IP-MS. [file 44154_2021_10_MOESM13_ESM.pdf]

Table S5. NPC, NE components, and factors involved in epigenetic regulation were identified in SWO1 IP-MS

Protein	Replicate I		Replicate II		Replicate III	
	Unique Peptide	Score	Unique Peptide	Score	Unique Peptide	Score
SWO1	51	6817	98	6623	107	8068
Nup98a	NA	NA	3	45	3	65
Nup54	NA	NA	NA	NA	2	23
Nup96	NA	NA	NA	NA	3	70
Nup155	NA	NA	1	30	NA	NA
Nup35	1	16	NA	NA	NA	NA
RAE1	NA	NA	1	24	NA	NA
LOS4	NA	NA	1	44	NA	NA
THO2	NA	NA	3	60	NA	NA
LINC4	NA	NA	6	90	6	59
SUN1	NA	NA	2	24	NA	NA
WIT1	2	17	NA	NA	1	37
UBP12	NA	NA	4	40	NA	NA
UBP13	NA	NA	NA	NA	2	63
CHR11	NA	NA	4	79	4	58
